# Explainable Split-Learning-Based Framework for Accurate Pulmonary Nodule Classification

**DOI:** 10.3390/bioengineering13050552

**Published:** 2026-05-13

**Authors:** Amira Bouamrane, Makhlouf Derdour, Ahmed Alksas, Norah Saleh ALghamdi, Mohamed Ghazal, Ayman El-Baz

**Affiliations:** 1 LIAOA Laboratory, Department of Computer Science, University of Souk Ahras, Souk Ahras 41000, Algeria; 2 LIAOA Laboratory, University of Oum El-Bouaghi-Larbi Benmhidi, Oum El-Bouaghi 04000, Algeria; 3 Department of Bioengineering, University of Louisville, Louisville, KY 40292, USA; 4 Department of Computer Sciences, College of Computer and Information Sciences, Princess Nourah Bint Abdulrahman University, Riyadh 11671, Saudi Arabia; 5 Department of Electrical, Computer, and Biomedical Engineering, University of Abu Dhabi, Abu Dhabi 59911, United Arab Emirates; 6 Research Institute for AI and Emerging Technology, Liwa University, Abu Dhabi 41009, United Arab Emirates

**Keywords:** lung cancer, diagnosis, CT scan, explainability, ResNet-50, split learning, XAI

## Abstract

Lung cancer rates are the highest among cancers, making it the leading cause of death worldwide. With advances in new technologies and diverse diagnostic methods, Computer-Aided Diagnosis Systems (CADx) have improved pulmonary nodule classification with notable accuracy and speed. However, limited data availability and privacy concerns remain significant challenges, in addition to the reported rates of false negatives and false positives. This work aims to develop an approach based on collaborative feature extraction between multiple centers, thus achieving data efficiency and diversity while ensuring privacy and reducing false positives and false negatives. This work proposes a new explainable feature-based split learning approach using diverse Computed Tomography (CT) scan datasets to evaluate data diversity and privacy. It adopts a split ResNet-50 architecture on the client side for feature extraction. On the server side, a hybrid 2D-CNN combined with an attention mechanism is used for final classification and decision-making. The architecture was evaluated using two ablation studies based on ConvNeXt-Tiny and EfficientNetB0. In addition, the model was tested on two external datasets to assess its robustness and generalizability, and with Local Interpretable Model-agnostic Explanations (LIMEs) and Grad-CAM to assess trustworthiness. This proposed approach showed an accuracy and F1-score of 99.38%, with a 1.23% false negative rate and zero false positives. Moreover, when tested on totally unseen datasets, the approach achieved an accuracy and an F1-score of 99.28% on the first dataset, with 1.24% false negatives and 0% false positives. In addition, when tested on the second dataset, the results indicate an ability to generalize, with 95.74% accuracy, with false negative and false positive rates of 7.07% and 1.41%, respectively.

## 1. Introduction

Lung cancer cells growing uncontrollably and their fast metastasizing to other parts of the body is considered the main reason for the high mortality rate caused by this disease [1,2], making early diagnosis challenging, which is crucial to reducing mortality [3]. Computed Tomography (CT) is conventionally regarded as the optimal imaging modality for identifying lung cancer and enhancing survival rates. This technique is composed of multiple X-ray images and provides 3D high-resolution images that are able to identify small nodules [4]. However, limited expertise and radiologists’ fatigue from long hours of work lead to subjective and erroneous diagnoses and are time-consuming, especially with the possible artifacts of this modality [5,6]. Computer-Aided Diagnosis Systems (CADx) have proven effective in improving decision-making objectivity, diagnostic accuracy, and speed, notably through the design of new deep learning (DL) and machine learning algorithms [7,8]. CADx systems typically achieve accuracy ranging from 90% to 99%, with high AUC values in lung cancer classification tasks [9,10]. Consequently, data quality and size are two essential criteria for ensuring the extraction of suitable and sufficient features, which improves the model stability and performance [11]. Therefore, researchers resort to combining several datasets from different sources or collaborating between various centers [12,13]. However, data security and confidentiality are crucial [14]. Federated learning (FL) facilitates collaboration between various centers without sharing data [15,16]. Despite these advances, existing approaches fail to simultaneously address data privacy, data heterogeneity, communication efficiency, and interpretability in multicenter lung cancer diagnosis [17,18]. Split learning (SL) addresses these challenges by partitioning the model into two parts. Within the institutional side, each institution or hospital conducts training using its data. Only intermediate features are shared with the server side, which then makes the predictions.

This paper proposes a new explainable split-learning modular architecture for lung cancer diagnosis, facilitating secure collaboration between clients with optimized communication. It is designed to ensure data heterogeneity, eliminate biases for generalizability, and offer high performance with flexible and dynamic client contributions, using a highly efficient pre-trained model to capture diverse pertinent and informative features from CT datasets. Simultaneously, the server utilizes a hybrid 2D-CNN with an attention mechanism to improve feature aggregation while also enhancing scalability and interpretability. In addition, Local Interpretable Model-agnostic Explanations (LIMEs) and Gradient-weighted Class Activation Mapping (Grad-CAM) were used to improve model interpretability.

## 2. Related Work

Several studies have proposed DL-based approaches for automated lung cancer detection and classification from CT scan images, particularly convolutional neural networks (CNNs), demonstrating promising performance across different datasets and architectures [7,8]. Shatnawi et al. established a CNN-based framework for the automated categorization of lung cancer utilizing the Chest CT-Scan Images dataset. The authors designed an eight-layer Enhanced CNN architecture and compared it with transfer learning models (InceptionV3, ConvNeXtSmall, EfficientNetB0, VGG16, and ResNet50), achieving 100% testing accuracy, outperforming InceptionV3 (76.9%), ConvNeXtSmall (87%), EfficientNetB0 (97.9%), and ResNet50 (94.5%) [19]. Priya et al. constructed a DL model (SE-ResNeXt-50-CNN) with the LUNA16 dataset. The SE-ResNeXt-50 architecture integrates ResNeXt’s multi-cardinality feature learning with Squeeze-and-Excitation (SE) channel attention to adjust feature significance, together with a CNN classifier for conclusive categorization. The SE-ResNeXt-50-CNN attained an accuracy, sensitivity, and an F1-Score of 99.15%, 97.58%, and 98.54% [20]. Klangbunrueang et al. designed a deep learning model utilizing VGG16, alongside a comparative analysis with ResNet50, InceptionV3, and MobileNetV2 architectures. VGG16 attained the highest performance with a test accuracy of 98.18% [21]. Additionally, Pahlevani et al. proposed a noise-aware CAD system for CT-based lung cancer detection, built on a modified InceptionV3 architecture augmented with Multi-Spatial Channel Attention (MSCA), achieving 98.94% accuracy, 99.17% specificity, and a 0.992 AUC [22]. Majumder et al. developed MENet, using Xception, InceptionResNetV2, and MobileNetV2, reaching 99.54% accuracy and a 99.10% F1-score on the IQ-OTH/NCCD dataset, and demonstrated strong generalizability on the LIDC-IDRI with an accuracy of 95.75% [23], while other researchers also used the LIDC-IDRI dataset to assess their proposed approaches [9,24,25]. Zheng et al. created an attention-ResNet-50 approach for lung cancer classification based on the LIDC-IDRI dataset. This model integrated Grad-CAM and includes multi-scale and multi-view features, as well as an attention mechanism, reaching an accuracy of 90.11% and an AUC of 95.66% [9], while Tang et al. introduced Lung-YOLO, a robust model incorporating a Cross-layer Aggregation Module (CLAM) and a Multiscale Dual-branch Attention (MSDA) mechanism, achieving 97% accuracy and 96.5% precision on the LUNA16 dataset, and 95.1% accuracy with 94.3% precision on the LIDC-IDRI dataset [26].

Despite these models demonstrating promising performance, their dependency on a single dataset can introduce bias and limit their generalizability. In contrast, federated-learning-based models have proven effective in addressing this limitation by enabling collaboration across multiple centers, consequently enhancing model robustness and addressing the problem of limited data availability. Usharani et al. developed FedLRes, a federated-learning-based model for automated lung cancer detection using the ResNet50 and IQ-OTH/NCCD CT dataset based on four clients for local training using ResNet50, followed by server-side aggregation (FedAvg) to update a global model. The FedLRes model reached an accuracy, sensitivity, and specificity of 99.40%, 99.03%, 99.36%, respectively, and 98.92% precision [27]. In addition, Xu et al. developed a heterogeneous federated learning model (HFLM) to predict non-small-cell lung cancer (NSCLC) using multi-center CT data. The proposed framework combined heterogeneous federated learning with a robust feature transfer strategy using VGG-16 as a local model and ResNet-18 as a global model. Mann–Whitney feature selection and mRMR were used for feature extraction, and classified with a Sparse Bayesian Extreme Learning Machine. Results showed that HFLM achieved the best performance across four centers with AUCs of 0.863, 0.837, 0.846, and 0.847, while Grad-CAM was used to enhance the model [28]. Moreover, Al-Shah et al. introduced an explainable Shared Generator-Based Serverless Federated Learning (SGS-FL) to enhance data privacy, heterogeneity, and scalability challenges for lung cancer diagnosis. Using LIDC-IDRI, NODE21, and NSCLC datasets, 92.5% accuracy and an AUC of 94.6% were achieved [29].

Despite these models helping address data scarcity and privacy issues to some extent, they also introduce other challenges, such as high demand for computational resources and communication overhead. Moreover, another challenge faced by AI-based diagnostic models in general is the issue of credibility and trust among clinicians, mainly due to the black-box nature of many AI systems. Therefore, Nady et al. developed ARXAF-Net, an explainable active reinforcement deep learning framework for lung cancer detection from CT images using Grad-CAM. This approach integrates active learning (AL) and reinforcement learning (RL) to address limited labeled data, followed by hybrid feature extraction combining traditional handcrafted features (GLCM, LBP, shape, intensity) with deep CNN features using attention fusion. The model performance improved from an initial 67% test accuracy (AUC = 75.1%) to 99.5% test accuracy and an AUC of 99.8%, with precision 99.9%, recall 98.4%, and F1-score 98.4% [10].

Table 1 summarizes the approaches employed in each study, including the datasets utilized, as well as results achieved, strengths, and limitations of each methodology. These approaches demonstrate the significance of verifying models on unseen samples and datasets. The requirement for larger datasets can be particularly difficult, especially when there is limited data availability, because depending on a single dataset can potentially induce bias. Therefore, data collaboration is considered to be a viable solution. Despite the current approaches that employ federated learning to achieve high diagnosis rates and solve the limited data availability, they face significant limitations, such as communication overhead and increased complexity, privacy challenges during the update exchange, scalability, and the need for specific computational resources, which may be challenging when adding more clients. Additionally, these models lack credibility and understanding. Although these studies used CNN models and deep learning techniques and achieved promising results, their internal decision-making processes remain difficult to explain or interpret. The key contributions of this study are as follows:

1. **Distributed, Secured Approach:**
  The split learning method enhances data privacy, where each client trains locally separately on its dataset until a specific split point for feature extraction, where clients send only the intermediate feature representations to the server, and the server uses the rest of the network, combined with additional layers for classification.
2. **Modular Split Learning Architecture:**
  The suggested architecture facilitates the effortless integration of new clients, hence improving processing efficiency and scalability.
3. **Overhead Reduced Bandwidth Communication:**
  This strategy was trained on the large-scale ImageNet dataset using a frozen ResNet50 to optimize data transmission between clients and the server, minimizing bandwidth usage and update exchanges.
4. **Hybrid Based on 2D-CNN with Attention Mechanism:**
  In order to process the upcoming extracted characteristics and identify spatial hierarchies and patterns in the data, the server employed a 2D-CNN with an attention mechanism to concentrate on the most pertinent information.
5. **Ablation Study with Alternative Backbones:**
  The proposed architecture was further evaluated through ablation studies using EfficientNetB0 and ConvNeXt-Tiny backbones, in addition to ResNet50, to assess the impact of backbone selection on model performance.
6. **Generalizability Assessment:**
  Evaluating the model performance on several external datasets provides insight into the proposed model’s robustness and generalizability.
7. **Explainable Trustworthy Approach:**
  To make the approach transparent and understandable, LIME and Grad-CAM were used to show the impact of specific features on the model’s decisions.

## 3. Methodology and Experiments

**Figure 1:**
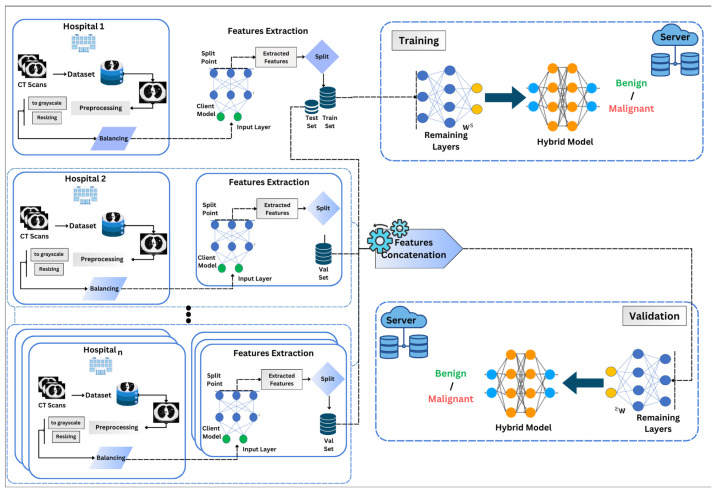
Simplified illustration of the proposed multi-center learning architecture for tumor classification in lung cancer. Clients use the first 170 layers of the pretrained ResNet-50 model to perform local CT image preprocessing and feature extraction. Only features are shared with the server, improving data privacy by avoiding direct image sharing. The server trains on LIDC-IDRI features, validates using features from all clients, and completes classification with the remaining network layers and an attention-based hybrid model.

Figure 1 shows the architecture of our approach. After dataset preprocessing, the clients use a pretrained ResNet-50 model, with ImageNet weights and frozen parameters (trainable = False). By learning rich, varied features from large-scale datasets such as ImageNet, this model can identify tumor attributes such as size, location, and structure. Moreover, only the first 170 layers of ResNet-50 (defined by split point = 170) are used to extract intermediate features. We tested using a smaller number of layers, which led to a decrease in accuracy, while a larger number of layers increased complexity. By utilizing this pretrained model on the client side, we limit training to the server side only, thereby reducing communication overhead between clients. Furthermore, this setup follows a decentralized approach in terms of data, inspired by split learning, specifically a feature-based split learning variant, which focuses on collaborative feature extraction, as shown in Figure 1. The intermediate features were then passed to the server, which used 60% of the first dataset for training. At the same time, the remaining 40% was concatenated with the extracted features from the other three clients for validation. The server processed these features using the remaining layers of ResNet50 and applied a 2D-CNN with an attention mechanism to perform the classification.

### 3.1. Datasets

#### 3.1.1. LIDC-IDRI Dataset

This study used a lung nodule-diagnosis subset of the Lung Image Database Consortium and Image Database Resource Initiative (LIDC-IDRI) dataset [30,31]. Thus, it has 15,548 nodules, 7992 of which are malignant and 7556 benign.

#### 3.1.2. The IQ-OTH/NCCD

The lung cancer dataset for the IQ-OTH/NCCD study was curated at the Iraq Oncology Teaching Hospital/National Cancer Center over a period of three months during autumn 2019. We used only the benign and malignant images from the datasets [32].

#### 3.1.3. Pre-Processing

**Figure 2:**
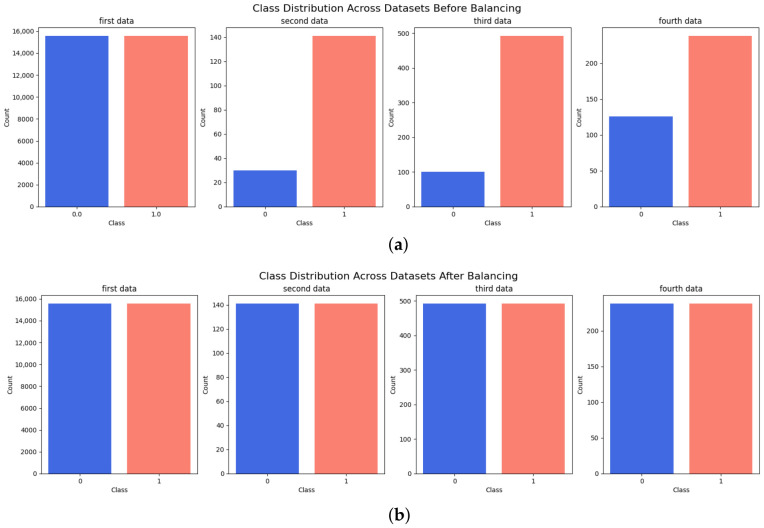
Class Distribution Across Datasets: Pre- and post-balancing distributions of classes in four datasets. (**a**) Before Balancing. (**b**) After Balancing.

We employed four different datasets, the LIDC-IDRI and three Kaggle datasets [33,34,35], to train and evaluate the model. To mitigate the risk of aggressive augmentation, we carefully constrained the augmentation strategy to transformations that preserve the anatomical and radiological characteristics of nodules, specifically, by applying moderate rotations, shifts, shearing, zooming, and horizontal flips to augment the minority class in the data set. For the third and fourth datasets, 162 images were reserved exclusively for testing before applying data augmentation or performing the training/validation split. These images were not used during either training or validation. This was necessary because augmentation was applied only to the remaining training/validation data, thereby preventing augmented versions or related samples from being indirectly exposed to the model. This procedure ensured that the internal test samples remained completely unseen by the model. Moreover, two additional datasets were used for external testing. The IQ-OTH/NCCD dataset was augmented using the ImageDataGenerator as shown in Figure 2. In contrast, for the DLCT dataset [36], we applied down-sampling. All images were scaled to 224 by 224 pixels to conform to the model’s input specifications, reducing computational complexity while preserving key features.

### 3.2. The Used Model for the SL Approach

#### 3.2.1. Client Side

**Figure 3:**
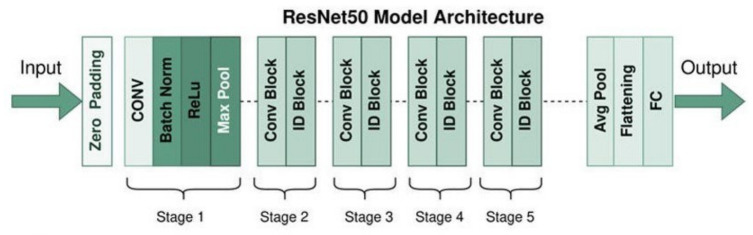
ResNet50 Model Architecture.

ResNet-50 addresses the vanishing gradient problem using residual blocks and shortcuts. It includes max pooling, convolutional layers with 3 × 3 filters, four residual blocks, global average pooling, and a final classification layer using softmax activation. Figure 3 [37].

#### 3.2.2. Server Side

On the server side, the model starts with the remaining layers of ResNet50 and then evaluates four different lightweight CNN configurations.

**Figure 4:**
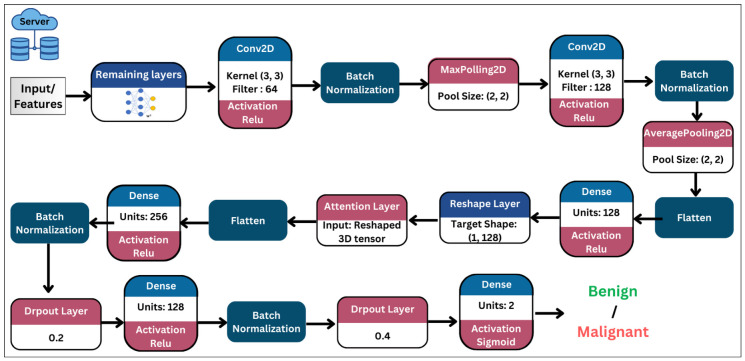
Server Model (Proposed CNN Architecture with an attention mechanism).

1. **2D-CNN and Hybrid 2D-CNN with Attention Mechanism:** The model using 2D-CNN with and without the attention starts with a Conv2D layer followed by BatchNormalization and MaxPooling2D for dimensionality reduction. Then, the Conv2D layer, BatchNormalization, and AveragePooling2D layers is used to further process features. After flattening, a dense layer captures complex features, and the data are reshaped for a self-attention layer. Furthermore, the hybrid variant incorporates a self-attention module to emphasize discriminative regions before classification. Post-attention, additional dense layers, batch normalization, and dropout layers are included for refinement and prevention of overfitting. The final output layer, using a sigmoid activation, generates the binary classification prediction. The hybrid model’s architecture is shown in Figure 4.
2. **1D-CNN with and without Attention Mechanism:** Both models begin with a GlobalAveragePooling2D layer to reduce the 4D input to 3D, followed by reshaping for 1D convolutional processing. A Conv1D layer with 64 filters is applied, followed by BatchNormalization and GlobalAveragePooling1D. For the attention-based 1D-CNN, the tensor is further processed using a self-attention layer to emphasize crucial features. The resulting output is then flattened and passed through dense layers, each with BatchNormalization and dropout for regularization. Finally, a fully-connected output layer with a sigmoid activation generates the binary classification prediction.
3. **Self-Attention Mechanism:** The server-side model applies a self-attention mechanism to the intermediate feature maps extracted at the split-point layer of the backbone. The feature map of shape (H×W×C) is first reshaped into a sequence of spatial tokens (T×C), where T=H×W, enabling the model to capture long-range spatial dependencies across the feature map. Query, Key, and Value projections are computed from the same input representation X, and the self-attention operation is defined as:
  (1) $$\mathrm{Attention}\left(Q,K,V\right)=\mathrm{softmax}\left(\frac{QK^{⊤}}{\sqrt{d_{k}}}\right)V$$
  where $d_{k}$ is the dimensionality of the Key vectors, and division by $\sqrt{d_{k}}$ stabilizes the dot-product values during training. The resulting attention weights dynamically highlight the most discriminative spatial regions. In the proposed architecture, a single self-attention block is integrated before a GlobalAveragePooling1D layer, which compresses the attended sequence into a compact feature vector before the final dense classification layers.
4. **Hyper-parameters:** To achieve a high performance of the proposed system, we used several values of different hyperparameters. Finally, we identified the optimal hyperparameters to reach the best results in Table 2.

## 4. Results

The proposed model was evaluated across ten hyperparameter configurations, as shown in Table 2, where each trial achieved competitive performance. Overall, the results demonstrate a high predictive performance across accuracy, AUC, and F1-score metrics, with only minor variations in false positive and false negative rates between configurations.

The final hyperparameter set was selected based on a multi-criteria analysis of the experimental trials, including strong validation and test performance, low and stable loss values, and balanced sensitivity–specificity trade-offs, instead of reporting performance using a single metric. Particular attention was given to reducing overfitting and improving generalization capacity across the two external datasets.

Consequently, with additional refinements, the server employs an attention-based classification head trained using binary cross-entropy loss and the Adam optimizer (learning rate = $10^{-4}$). Training is conducted for up to 100 epochs with a ReduceLROnPlateau scheduler (factor = 0.2, patience = 3, minimum learning rate = $10^{-5}$), along with dropout and early stopping to reduce overfitting. To further reduce the risk of overfitting, the test samples were kept completely independent of the training and validation subsets, and data augmentation was applied only to the training data. Model performance was also evaluated on two external datasets that were not used during model development. The consistency of the results across internal and external testing, together with bootstrap confidence interval analysis, supports the robustness of the proposed framework and reduces the likelihood that the high AUC values resulted from overfitting. Both client-side and server-side training use a batch size of 64, and all experiments were conducted on Kaggle using a V100 GPU.

Table 3 summarizes a comprehensive comparison of ResNet50, EfficientNetB0, and ConvNeXt-Tiny backbones across 2D, 2D-Attention, 1D, and 1D-Attention configurations, reporting training/validation performance, test metrics, statistical confidence intervals, inference latency, and computational complexity.

### 4.1. Training and Validation Performance

**Figure 5:**
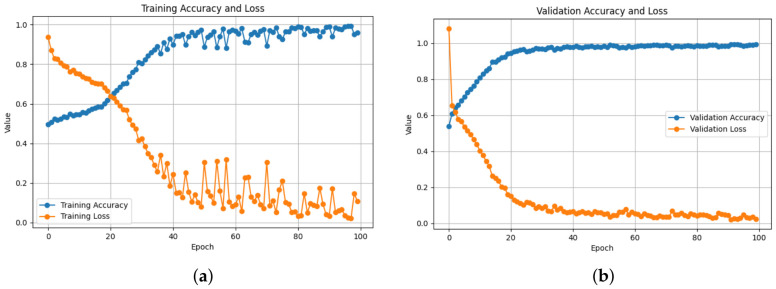
Hybrid Model with Attention: Training and Validation Performance. The figure presents the learning curves of the proposed hybrid model with an attention mechanism. (**a**) Training accuracy and training loss across epochs, showing an increased accuracy and loss reduction. (**b**) Validation accuracy and validation loss over epochs, demonstrating strong generalization and convergence.

**Figure 6:**
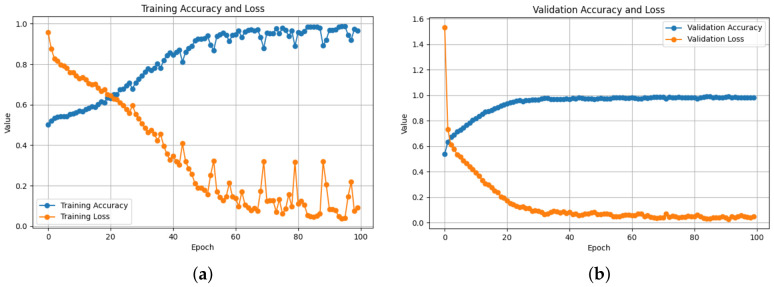
Training and Validation Performance of the 2D-CNN Model: (**a**) The training accuracy increases while the training loss decreases, which shows that the model is learning useful features. (**b**) After a certain number of epochs, the validation accuracy levels out at over 98.28%, and the validation loss drops to 0.047. This shows that the model can generalize.

**Figure 7:**
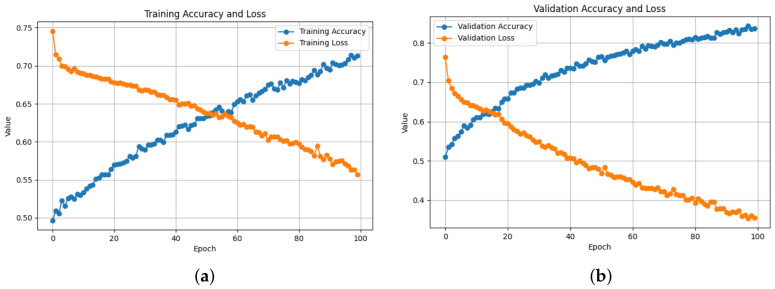
Training and Validation Performance of the 1D-CNN with Attention: Accuracy and loss curves over 100 epochs. (**a**) Training accuracy increases progressively up to 71.74%, while training loss decreases, reaching 0.5468, achieving the worst results among the four models. (**b**) Validation accuracy improves effectively, achieving 86.75%, with a corresponding reduction in validation loss to 0.3056, indicating lower capability of generalization.

**Figure 8:**
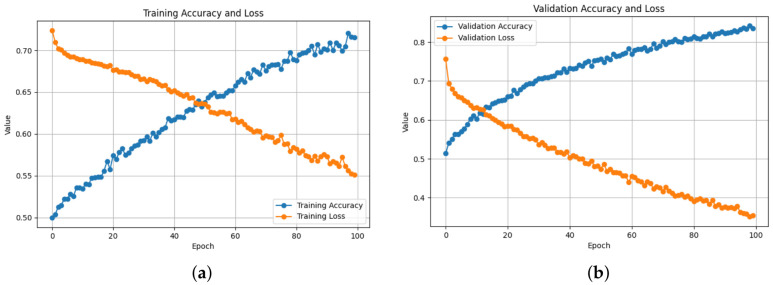
1D-CNN Training and Validation Performance: Training and validation accuracy and loss curves over 100 epochs. (**a**) The training phase demonstrated a gradual enhancement in accuracy, reaching 85.04%, accompanied by a reduction in loss to 0.1852, signifying effective learning. (**b**) Validation phase demonstrating consistent accuracy improvement of 92.43% and loss reduction, indicating good generalization without significant overfitting.

Training and validation behavior across all architectures demonstrates stable convergence and consistent learning. As shown in Table 3, the ResNet50-based 2D-Attention model achieved the best training performance with a loss of 0.0303 and accuracy of 99.09%, while keeping strong validation performance, with an accuracy of 98.89% and 0.0296 loss, as also illustrated in Figure 5. Meanwhile the loss increased to 0.1061 when removing the attention, as shown in Figure 6, with a training accuracy of 96.03%, while in validation it reached an accuracy of 98.28% with 0.0471 of loss. On the contrary, using 1D-CNN as shown in Figure 7 and Figure 8, removing the attention mechanism enhanced the performance from 0.5468 of loss and 71.74% of accuracy in training to 0.3293 and 85.04%, similarly in validation, from 0.3056 of loss and 86.75% of accuracy to 0.1852 and 92.43%. In the ablation study using ConvNeXt-Tiny as the backbone, this experiment achieved the lowest validation loss of 0.0095 with the 2D attention mechanism, indicating highly stable optimization behavior. In contrast, EfficientNetB0 achieved slightly higher loss values but remained consistent across runs. In the three backbones, attention-based 2D-CNN models significantly outperformed models using 1D-CNN, confirming the importance of spatial feature representation.

### 4.2. Classification Performance on the Internal Test Set

**Figure 9:**
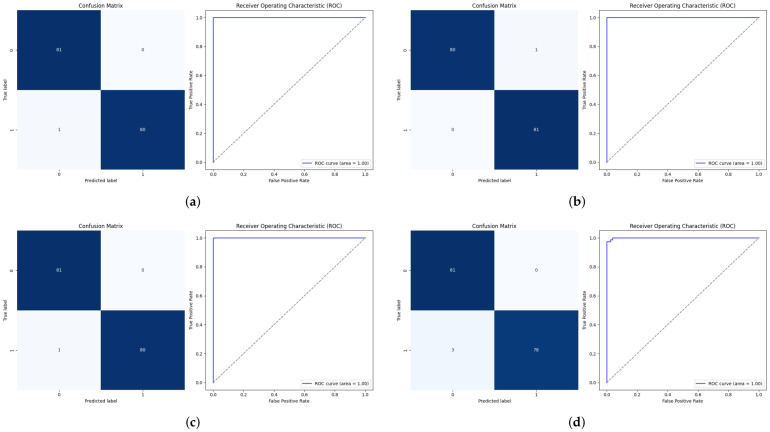
The confusion matrices illustrate the proposed model’s classification performance using 2D-CNN and 1D-CNN with and without an attention mechanism. (**a**) Hybrid Model. (**b**) 2D-CNN Model. (**c**) 1D-CNN with Attention Mechanism. (**d**) 1D-CNN Model.

The classification results on the internal test demonstrate that the proposed attention-based 2D-CNN showed the best results across all backbone architectures. Moreover, using ResNet50 as a backbone, both 2D-CNN and 1D-CNN with the attention mechanism achieved the highest results, with an accuracy of 99.38%, an F1-score of 99.38%, and an Area Under the Curve (AUC) of 100%, with zero false positives and very low false negative rates (1.23%). Figure 9a–d illustrate the performance of the proposed approach using ResNet50 across the four configurations using 2D-CNN and 1D-CNN, both with and without the attention mechanism. Similar trends were observed across EfficientNetB0 and ConvNeXt-Tiny, where attention improved sensitivity, precision, and overall classification balance, as shown in Table 4. The ablation using EfficientNetB0 with 1D-CNN- Attention and ConvNeXt-Tiny using 1D-CNN achieved the highest F1score of 99.38%. Although some non-attention models achieved competitive accuracy, they showed higher variability in false positive and false negative distributions.

### 4.3. Statistical Validation

Robustness was evaluated using bootstrap resampling (N = 1000), as illustrated in Table 3. The ResNet50-based 2D-CNN-Attention model achieved highly stable confidence intervals for both AUC and accuracy, with an AUC 95% CI of [100, 100] and an accuracy 95% CI of [98.15, 100]. Other ResNet50-based configurations also demonstrated strong statistical stability, with several intervals reaching [100, 100]. ConvNeXt-Tiny showed strong statistical reliability in several configurations, whereas EfficientNetB0 showed comparatively wider confidence intervals, indicating higher sensitivity to data variability. Overall, these results support the statistical stability and robustness of the proposed attention-based framework across different sampling conditions.

### 4.4. Inference Latency and Computational Efficiency

As shown in Table 3, the computational efficiency analysis highlights clear trade-offs between accuracy and efficiency across architectures. ResNet50-based models exhibited moderate inference latency, with mean values around 15–61 ms per image depending on configuration. EfficientNetB0 provided the most efficient inference profile in lightweight configurations, achieving median latency as low as 7.59 ms/image in the 1D variant. Moreover, ConvNeXt-Tiny achieved strong performance but with a higher computational cost in 2D-CNN (GFLOPs up to 0.0942 and 238 s training epoch time), while keeping competitive throughput in optimized variants. Across all architectures, 1D models reduced computational complexity (down to 0.0003 GFLOPs) but at the cost of significantly lower predictive performance. On the contrary, 2D-CNN with an attention mechanism provided the best balance between accuracy, robustness, and computational cost. Throughput analysis further confirms this trade-off: EfficientNetB0 achieved the highest throughput in optimized configurations (up to 143.9 images/s), while ResNet50 and ConvNeXt-Tiny maintained balanced but slightly lower processing rates.

Attention-based 2D models outperform 1D variants across all backbones, with ResNet50 achieving the best overall performance, ConvNeXt-Tiny showing strong stability, and EfficientNetB0 offering the best efficiency–performance trade-off, while attention improves key classification metrics and efficiency is maintained across configurations.

## 5. Discussion

The experimental results demonstrate that the proposed attention-based deep learning framework achieved high performance across all evaluated backbones and architectural variants. Across ResNet50, EfficientNetB0, and ConvNeXt-Tiny, the integration of attention mechanisms within 2D-CNN architectures improves classification performance, stability, and generalization ability compared to both non-attention counterparts and 1D-CNN. Moreover, a key observation is the strong balance between performance and computational efficiency. While 1D-CNN variants significantly reduce computational cost (which decreased to 0.0003 GFLOPs), this reduction is associated with degraded classification performance, particularly in sensitivity and F1-score, while 2D-CNN architectures kept more informative and richer spatial representations, leading to superior discrimination capability across all datasets. Furthermore, ResNet50-based and ConvNeXt-Tiny models achieved the most balanced performance, a high accuracy of 99.38%, AUC of 100% using ResNet50-based attention, and 99.39% using ConvNeXt-Tiny with 1D-CNN, and strong statistical stability as confirmed by bootstrap confidence intervals. While EfficientNetB0 showed less stability, it provided the best balance of efficiency and performance, achieving the highest throughput (up to 143.9 images/s) with minimal computational overhead.

### 5.1. Backbone and Architectural Analysis

The comparative analysis across backbones demonstrates that architectural depth and representational capacity strongly influence model performance. ResNet50 consistently achieved the highest test accuracy with the most stable convergence, especially when using a 2D-CNN with an attention mechanism, indicating that residual learning effectively enhances deep feature extraction from medical images. Moreover, ConvNeXt-Tiny demonstrates comparable validation performance, achieving the lowest validation loss (0.0095), suggesting strong optimization stability. Despite this, its computational cost is higher in 2D-CNN, reaching up to 0.0942 GFLOPs, indicating a balance between performance and efficiency. On the contrary, EfficientNetB0 showed lower predictive performance, but with significantly improved computational efficiency. Its lightweight design allows for high throughput and reduced latency, making it particularly suitable for real-time or resource-constrained cases.

### 5.2. Attention Mechanism Contribution

The integration of the attention mechanism is a critical factor that improved the performance across the three ablations. Across all backbones, the attention-based models achieved higher accuracy, sensitivity, precision, and F1-score and simultaneously reduced false positive and false negative rates. In ResNet50, using 2D-CNN with an attention mechanism achieved an accuracy of 99.38% and an AUC of 100%, with a complete balance of sensitivity and specificity. A similar trend is observed in ConvNeXt-Tiny and EfficientNetB0, indicating that attention mechanisms enhance feature selection by focusing on the most relevant regions while ignoring redundant or noisy features. Moreover, the statistical validation using bootstrap confidence intervals confirms that the ResNet50-based attention model showed strong statistical stability, with an AUC 95% CI of [100, 100] and an accuracy 95% CI of [98.15, 100], indicating that attention not only improves mean performance but also reduces uncertainty, which is essential for medical AI CADx.

### 5.3. Generalization and External Dataset Robustness

Additionally, when compared with the studies in the related work (Table 1), the proposed model outperformed most of the models reported, except for the model proposed by Usharani et al., who used ResNet50 based on the IQ-OTH/NCCD dataset and achieved an accuracy of 99.40% [27], and the model proposed by Nady et al., which achieved an accuracy of 99.5% [10]. However, these models suffer from a lack of explainability. Moreover, the model proposed by Majumder also achieved higher results with an accuracy of 99.54% [23]. Additionally, the study indicates the model still suffers from the problem of false negatives and false positives, as well as the challenge of the high heterogeneity of the dataset. These findings emphasize the importance of employing effective feature extraction techniques, addressing class imbalance, and managing high complexity, in addition to training models on data from diverse sources to improve their robustness and generalizability.

**Figure 10:**
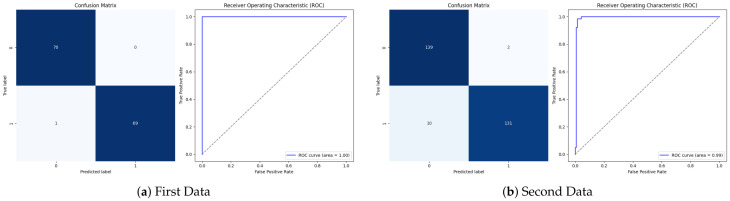
Proposed Approach using 2D-CNN with Attention Across External Datasets: Performance evaluation of the proposed 2D-CNN with attention mechanism on two external datasets. Each subfigure shows the confusion matrix as well as the ROC curve for (**a**) the first dataset and (**b**) the second dataset. The model achieves accuracies of 99.28% on the first dataset and 95.74% on the second one, with sensitivities of 98.57% and 92.90%, respectively. Specificity and accuracy remain at high levels (up to 100%), with AUC values of 100% and 99.23%, thus demonstrating robust generalization across independent data sources.

Moreover, the absence of evaluation results on external datasets is a shortcoming in most previous studies. Therefore, the proposed approach using ResNet50 as backbone and 2D-CNN-Attention was tested using two different external datasets, as shown in Figure 10a,b, and also compared to the other three models, 2D-CNN without attention mechanism, and 1D-CNN with and without attention, as shown in Table 4. Generally, 2D-CNN with attention achieved the best results on both datasets, with an accuracy of 99.28% on the first dataset and specificity, precision, and AUC of 100%. On the second dataset, it achieved the best results among the four models, with an accuracy of 95.74%, an F1-score of 95.62%, and an FPR of 1.43%. However, the two models using 2D-CNN and 1D-CNN with an attention achieved complete results on the first external dataset with 100% accuracy. However, the results were lower when tested on the second dataset. This is consistent with the loss results achieved by these three models during training and validation. This is contrary to the first model, which showed stability and robustness in all phases. Furthermore, the proposed model also outperformed the ablation study in the generalization experiments, where EfficientNetB0 and ConvNeXt-Tiny backbones using 1D-CNN and 2D-CNN architectures, both with and without an attention mechanism, were tested on the IQ-OTH/NCCD dataset. ConvNeXt-Tiny demonstrated high generalizability across all configurations, with 2D-CNN achieving 97–98% accuracy, along with well-balanced precision and recall. EfficientNetB0 2D-CNN achieved the lowest results, with accuracies of 73.75% and 50% with 100% of FNR using the other architectures, indicating difficulty in recognizing malignant cases.

These results show the proposed model’s effectiveness and its capability to maintain strong performance across various conditions and datasets. However, the external dataset on which the model was tested is relatively small, so the model will be tested in the future on larger and more diverse datasets.

#### Explanation Results

Four images were randomly selected from the dataset to analyze the predictions of the four models. LIME was configured with 1000 samples and five features, alongside Grad-CAM, to identify the key features and regions influencing the models’ decisions, across all four configurations (1D-CNN and 2D-CNN, with and without an attention mechanism), as presented in Table 5, Table 6 and Table 7.

### 5.4. LIME and Grad-CAM Explanations of ResNet50-Based Models

The four models correctly classified image 4 (True Label: 1) as malignant, with high confidence scores. The 2D-CNN with and without attention achieved a confidence of 1.000, the 1D-CNN with attention scored 0.757, and the 1D-CNN without attention scored 0.896, indicating strong model certainty in the malignant prediction. However, the four models showed inconsistent performance on image 3 (True Label: 1), which also contains a malignant tumor, where the 2D-CNN with attention achieved 0.935, the 2D-CNN without attention achieved 0.881, and the 1D-CNN with attention decreased significantly to 0.021 and the 1D-CNN without attention to 0.022, indicating low confidence. Moreover, as shown by LIME, the models did not precisely consider the tumor region important for classification, which could be a result of the non-solid nature of the nodule. For image 2 (True Label: 0), the 2D-CNN with attention and the 1D-CNN without attention misclassified it as malignant with low confidence scores of 0.062 and 0.316, respectively, while the 2D-CNN without attention and the 1D-CNN with attention correctly classified it as benign with confidence scores of 0.003 and 0.368, respectively. For image 1 (True Label: 0), all four models correctly predicted the benign label, with the 2D-CNN with attention showing a very low confidence of 0.024, the 2D-CNN without attention 0.047, the 1D-CNN with attention 0.829, and the 1D-CNN without attention 0.532. In most models across images 1 and 2, the tumor area was not considered the primary discriminative region; instead, attention was focused on parts of the lung’s outer wall or the upper lobes, as evidenced by both the LIME superpixel highlights and the Grad-CAM thermal overlays.

### 5.5. LIME and Grad-CAM Explanations of EfficientNetB0 and ConvNeXt-Tiny

Table 6 and Table 7 illustrate the results of applying explainable AI to the decisions of EfficientNetB0 and ConvNeXt-Tiny. Both EfficientNetB0 and ConvNeXt-Tiny backbones showed high performance in recognizing malignant cases, with ConvNeXt-Tiny showing higher confidence on image 4, which aligns with its performance during the training, validation, and testing phases compared to its EfficientNetB0 counterpart. However, ConvNeXt-Tiny, using 1D-CNN without an attention mechanism, was unable to recognize the tumor characteristics in image 3, unlike when using a 2D-CNN, where it correctly identified the malignant case with a confidence score over 0.99. EfficientNetB0 offered more balanced performance overall, correctly classifying all four models on both benign images, but with a low confidence score. Furthermore, ConvNeXt-Tiny produced more false positives on benign cases, particularly with the 1D-CNN without attention, achieving a high erroneous confidence of 0.741 on image 2. From the explainability-highlighted regions, neither backbone consistently localized activation to the nodule region. Both focused on peripheral lung structures, though ConvNeXt-Tiny showed slightly better spatial concentration in correctly classified malignant cases. In conclusion, ConvNeXt-Tiny demonstrates stronger sensitivity for malignant detection in 2D-CNN architectures, while EfficientNetB0 provides more reliable and balanced specificity across all model variants.

Despite the LIME method giving fast and local explanations, its accuracy may be low because, during the tuning of LIME, adjustments were made to only five features. This may impair the interpretability of the model because the LIME images provided focused only on a small subset of all the features. This constraint may partially explain the observations across all three backbones using ResNet50, EfficientNetB0, and ConvNeXt-Tiny, where LIME superpixels highlighted peripheral lung wall structures and non-nodule regions rather than the clinically relevant tumor area, such as image 3. The complementary use of Grad-CAM alongside LIME partially addressed this limitation, as the thermal activation overlays provided a broader gradient-based view of the discriminative regions used by the models, offering a more complete view of model decisions that LIME alone could not capture. However, the spatial disagreement between LIME and Grad-CAM highlighted regions, especially when using the 1D-CNN across all backbones, indicating that more future work is required to explore optimizing LIME’s sample size and feature configuration, or explore more explanatory techniques to improve the trustworthiness and reliability of the proposed model. Future work will also investigate multimodal imaging approaches, including PET-CT, to integrate anatomical and functional imaging information for more comprehensive lung cancer assessment.

Furthermore, despite the promising results of the proposed approach, several limitations still require further investigation, which will be addressed in future work, such as dataset size constraints, domain shift between datasets, and the computational cost of the hybrid architecture.

## 6. Conclusions

Given the global challenge of lung cancer and the search for effective diagnostic systems that allow for collaboration among various medical centers with scalability and flexibility, there is an additional challenge beyond accuracy, specifically protecting sensitive patient data. This requires a secure and modular system with minimal complexity and reduced communication overhead. In this study, we present the use of a novel Explainable Split Learning (SL) technique for lung cancer diagnosis that allows for involvement of multiple centers with minimal complexity, communication overhead, and utmost patient protection. The proposed technique has implemented ResNet-50 on the client side to extract quality features from the local data. At the same time, the server used a hybrid 2D-CNN with an attention mechanism, with the remaining layers of ResNet-50 for the classification. The proposed approach demonstrated strong predictive performance and generalizability for pulmonary nodule classification. Finally, LIME and Grad-CAM were utilized to assess the suggested model’s trustworthiness. However, more comprehensive research is necessary to evaluate the model’s applicability to a wider range of scenarios and to include a greater number of clients with varied datasets. Leveraging diverse medical imaging data and complementary information from multiple imaging sources has the potential to further enhance the performance while improving its robustness and clinical applicability.

## Figures and Tables

**Table 1:** Comparison of Related Works: The table summarizes and compares previous works, outlining each study reference, the model, dataset, publication year, achieved results, and reported limitations.

#	Study	Year	Approach	Dataset	Results (%)	Limitations
1	Usharani et al. [27]	2025	FL-Resnet	IQ-OTH/NCCD	Prec = 98.92% Acc = 99.40% Spec = 99.36%	Small dataset Data dependency Limited explainability Limited generalizability
2	Xu et al. [28]	2025	FL ResNet-18 VGG-16	multi-center CT data	AUCs of 0.863, 0.837, 0.846, and 0.847	High Complexity Class imbalance
3	Shatnawi et al. [19]	2025	ConvNeXtSmall VGG16 ResNet50 InceptionV3 EfficientNetB0	Chest CT-Scan Images dataset	Acc = 100%	Small homogenous dataset Potential overfitting Limited Generalizability and explainability
4	Zheng et al. [9]	2024	Attention-ResNet-50	LIDC-IDRI	Acc = 90.11 AUC = 95.66	Limited generalizability Data dependency
5	Tang et al. [26]	2025	Lung-YOLO	LIDC-IDRI LUNA16	LUNA16: Acc = 97.5 Prec = 96.5 Rec = 96.9 LIDC-IDRI: Acc = 95.1 Prec = 94.3 Rec = 93.4	Data dependency Limited explainability
6	Majumder et al. [23]	2024	Xception Inception ResNetV2 MobileNetV2	LIDC-IDRI IQ-OTH/NCCD	IQ-OTH/NCCD: Acc = 99.54 Prec = 99.62 Rec = 98.61 F1 = 99.10 LIDC-IDRI: Acc = 95.75 Prec = 92.36 Rec = 92.95	False positives/negatives
7	Al-Shah et al. [29]	2025	SGS-Federated Learning	LIDC-IDRI NODE21 (X-ray) NSCLC PET-CT	Acc = 92.5 Dice = 0.83 AUC = 0.946	Limited generalization
8	Priya et al. [20]	2025	SE-ResNeXt-50-CNN	LUNA 16	Acc = 99.15% Sens = 97.58% Prec = 99.51% Spec = 99.80% F1-score = 98.54%	High computational complexity limited dataset size Potential overfitting Limited explainability
9	Klangbunrueang et al. [21]	2025	ResNet50 InceptionV3 MobileNetV2 VGG16	IQ-OTH/NCCD	Acc = 98.18%	Limited Dataset
10	Nady et al. [10]	2026	ARXAF-Net-CNN	30,020 CT images	Acc = 99.5% AUC = 99.8% Prec = 99.9% Recall = 98.4% F1-score = 98.4%	Limiting generalizability
11	Halder [25]	2025	WaveLCDNet	LIDC-IDRI DSB2017	LIDC-IDRI: Accuracy = 96.70% Sensitivity = 96.89% Specificity = 95.52% Kaggle DSB2017: Accuracy = 95.90%	Limited clinical diversity
12	Pahlevani et al. [22]	2026	InceptionV3	IQ-OTH/NCCD	Acc = 98.94% Spec = 99.17% Sens = 98.46% AUC = 99.2%	Small and imbalanced Risk of overfitting Lack of external validation
13	Gautam et al. [24]	2024	ResNet-152 DenseNet-169 EfficientNet-B7	LIDC-IDRI	Acc = 98.74/97.23 F1 = 98.33 Prec = 98.58 Sens = 98.07 AUC = 94.68	High computational cost Data dependency

**Table 2:** Hyperparameters and Performance of the 10 Different Trials: This table summarizes the results achieved by the proposed model during the training, validation, and test phases across a set of experiments using different hyperparameter values.

Trial	01	02	03	04	05	06	07	08	09	10
Learning rate (%)	0.46276	0.60809	0.088876	0.14195	0.66633	0.44921	0.1382	0.29124	0.06047	0.089102
Dropout_1_	0.2	0.2	0.4	0.2	0.2	0.3	0.3	0.4	0.3	0.4
Dropout_2_	0.4	0.3	0.3	0.3	0.3	0.4	0.2	0.3	0.4	0.3
Dense_1_ units	256	128	256	512	128	128	512	384	256	256
Dense_2_ units	128	64	192	64	64	64	192	128	128	192
Training Accuracy (%)	97.39	96.74	97.26	95.89	96.80	97.26	93.66	96.46	97.34	97.63
Training Loss	0.077	0.092	0.070	0.120	0.087	0.072	0.160	0.0956	0.0736	0.0705
Validation Accuracy (%)	98.55	97.75	98.03	98.32	97.85	98.14	98.46	98.70	96.78	98.49
Validation Loss	0.0501	0.0623	0.0524	0.0465	0.0620	0.0489	0.0487	0.0364	0.0864	0.0412
Test Accuracy (%)	100	96.91	97.53	96.91	91.97	98.14	100	97.53	98.76	96.29
False Positives (%)	0	2.70	1.23	2.46	12.34	0	0	2.46	1.23	3.70
False Negatives (%)	0	2.46	3.70	3.70	3.70	3.70	0	2.46	1.23	3.70
Sensitivity (%)	100	97.56	96.29	96.29	96.29	96.29	100	97.53	98.76	96.29
Specificity (%)	100	96.29	98.76	97.53	87.65	100	100	97.53	98.76	96.29
Precision (%)	100	96.34	98.73	97.50	88.63	100	100	97.53	98.76	96.29
AUC (%)	100	99.74	99.87	99.86	94.48	99.95	100	97.53	98.76	96.29
F1-score (%)	100	96.93	97.60	96.89	92.30	98.13	100	97.53	98.76	96.29

**Table 3:** Comparison of the models’ performance across backbones and architectural variants.

Metric	ResNet50 (Main)				EfficientNetB0 (Ablation)				ConvNeXt-Tiny (Ablation)			
	2D + Attn	2D	1D + Attn	1D	2D + Attn	2D	1D + Attn	1D	2D + Attn	2D	1D + Attn	1D
*Training & Validation*												
Training Loss	0.0303	0.1061	0.5468	0.3293	0.0868	0.1677	0.5144	0.5121	**0.0289**	0.1026	0.4277	0.3926
Training Accuracy (%)	99.09	96.03	71.74	85.04	96.94	94.68	74.77	74.98	**99.08**	97.09	79.87	82.22
Validation Loss	**0.0296**	0.0471	0.3056	0.1852	0.0821	0.1031	0.3413	0.3596	0.0095	0.1024	0.1658	0.1653
Validation Accuracy (%)	98.89	98.28	86.75	92.43	96.85	95.78	81.26	80.00	**99.64**	99.68	93.08	93.31
*Classification Performance (Test Set)*												
Prediction Accuracy (%)	**99.38**	**99.38**	**99.38**	98.14	98.77	95.68	**99.38**	**99.38**	93.21	94.44	98.77	**99.38**
False Positives (%)	**0**	1.23	**0**	**0**	2.47	4.94	1.23	**0**	11.11	4.94	2.47	1.23
False Negatives (%)	1.23	**0**	1.23	3.70	**0**	3.70	**0**	1.23	2.47	6.17	**0**	**0**
Sensitivity (%)	98.76	**100**	98.76	96.29	**100**	96.30	**100**	98.77	97.53	93.83	**100**	**100**
Specificity (%)	**100**	98.76	**100**	**100**	97.53	95.06	98.77	**100**	88.89	95.06	97.53	98.77
Precision (%)	**100**	98.78	**100**	**100**	97.59	95.12	98.78	**100**	89.77	95.00	97.59	98.78
AUC (%)	**100**	99.99	**100**	99.92	99.74	99.21	**100**	**100**	98.19	98.61	99.02	99.98
F1-score (%)	**99.38**	**99.38**	**99.38**	98.11	98.78	95.71	**99.39**	99.38	93.49	94.41	98.78	**99.39**
*Statistical Validation—Bootstrap 95% CI (N=1000)*												
AUC 95% CI	[100, 100] ^†^	[100, 100] ^†^	[100, 100] ^†^	[100, 100] ^†^	[99.04, 100]	[98.08, 99.88]	[100, 100] ^†^	[100, 100] ^†^	[96.54, 99.40]	[96.57, 99.85]	[96.93, 100]	[99.91, 100]
Acc 95% CI	[98.15, 100]	[100, 100] ^†^	[100, 100] ^†^	[96.91, 100]	[96.91, 100]	[91.98, 98.15]	[98.15, 100]	[98.15, 100]	[89.51, 96.91]	[90.74, 97.53]	[96.91, 100]	[98.15, 100]
*Inference Latency*												
Mean (ms/img)	15.15	20.12	61.62	53.41	16.10	15.46	54.11	**7.59**	20.16	17.23	57.38	**7.65**
Median (ms/img)	15.05	19.84	60.75	53.41	15.96	15.20	53.40	**7.52**	19.39	17.15	57.60	**7.66**
P95 (ms/img)	15.81	21.58	70.13	**53.41**	17.08	17.63	59.71	**8.24**	24.35	17.87	60.54	**8.00**
Latency Std (ms)	0.32	1.29	4.55	**0.00**	0.58	0.90	3.13	**0.31**	2.25	0.43	2.48	**0.17**
*Training Throughput & Epoch Time*												
Train Epoch (s)	107.04	120.39	83.98	**75.62**	134.45	131.83	98.28	97.16	238.31	243.95	185.78	172.41
Val Epoch (s)	64.65	73.75	50.15	**46.42**	82.43	81.63	60.00	59.27	145.85	149.03	110.90	104.48
Throughput (img/s)	101.7	90.4	129.6	**143.9**	80.9	82.6	110.7	112.0	45.7	44.6	58.6	63.1
*Computational Complexity*												
GFLOPs	0.0304	0.0304	**0.0003**	**0.0003**	0.0394	0.0394	**0.0003**	**0.0003**	0.0942	0.0941	**0.0003**	**0.0003**
MACs	15,190,371	15,190,112	**127,843**	**127,712**	19,706,211	19,705,952	**162,483**	**162,352**	47,091,019	47,070,080	**162,483**	**128,224**
Total Params	437,185	437,185	**115,265**	**115,265**	529,345	529,345	145,985	145,985	363,457	363,457	145,985	**90,689**

*Note:* Bold indicates the selected best-performing model at each metric. The † indicates the highest confidence intervals.

**Table 4:** Performance Comparison of Models Across Two External Datasets.

Metric	2D-CNN-Attention		2D-CNN		1D-CNN-Attention		1D-CNN	
	Data 1	Data 2	Data 1	Data 2	Data 1	Data 2	Data 1	Data 2
Accuracy (%)	99.28	95.74	100	93.97	100	87.23	99.28	85.81
FPR (%)	0	1.43	0	11.34	0	12.76	0	22.69
FNR (%)	1.42	7.07	0	0.70	0	12.76	1.42	5.67
Sensitivity (%)	98.57	92.90	100	99.29	100	87.23	98.57	94.32
Specificity (%)	100	98.56	100	88.65	100	87.23	100	77.30
Precision (%)	100	98.49	100	89.74	100	87.23	100	80.60
AUC (%)	100	99.23	100	99.40	100	93.85	99.95	94.21
F1-score (%)	99.28	95.62	100	94.27	100	87.23	99.28	86.92

**Table 5:** LIME and Grad-CAM explanations generated by four lightweight CNN variants using **ResNet50** as backbone, applied to four representative CT images.

Original Image&True Label	XAI	2D-CNN Attention	2D-CNN Without Att	1D-CNN Attention	1D-CNN Without Att
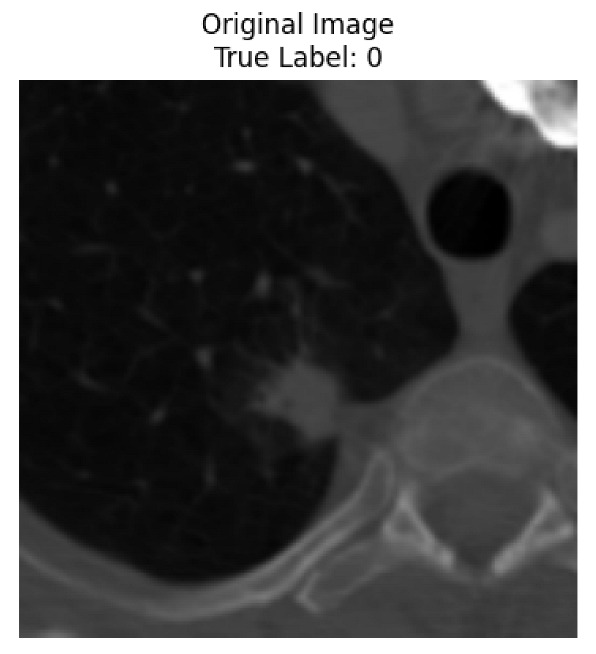	LIME	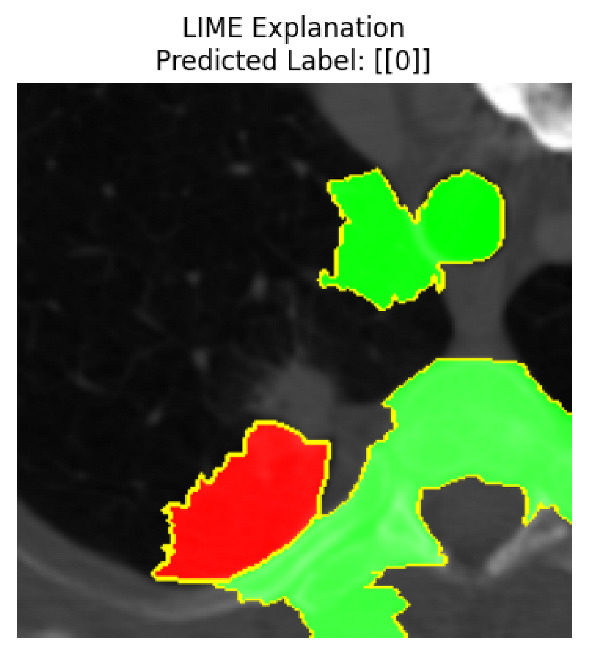	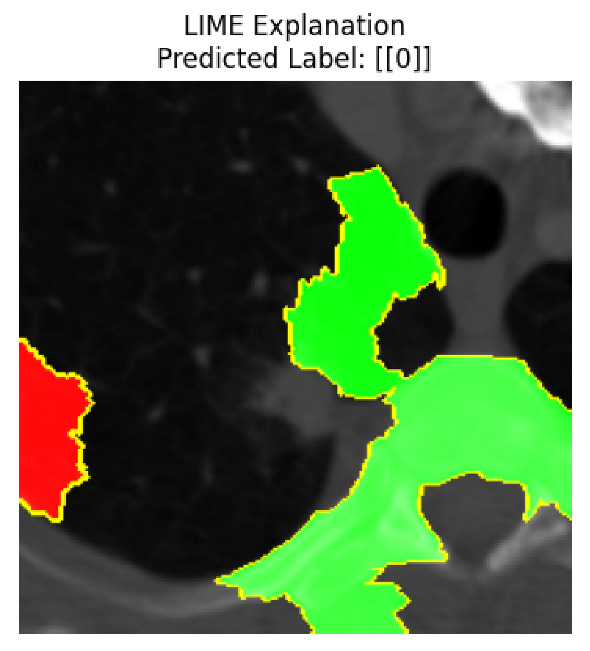	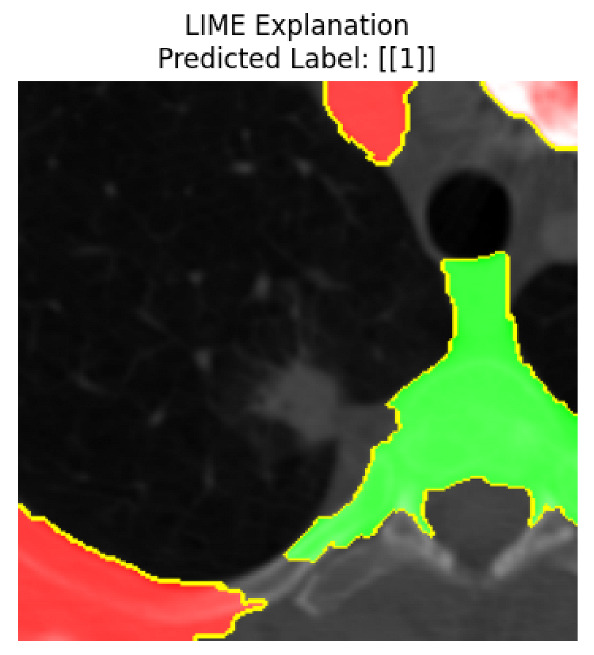	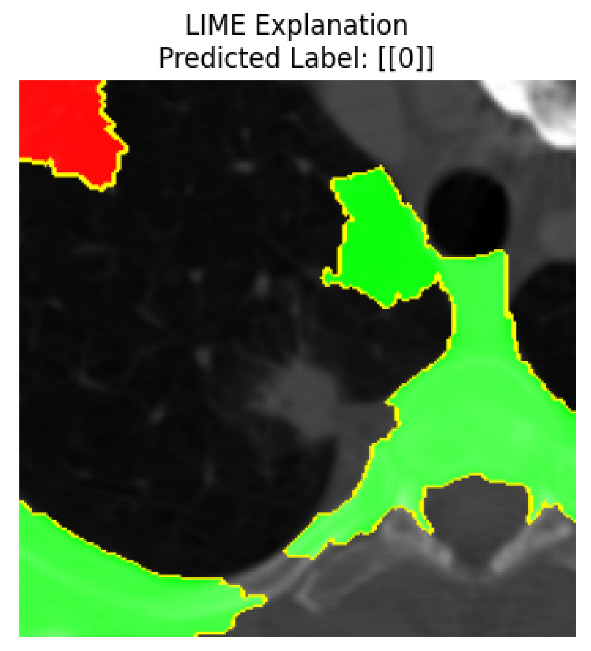
	Grad-CAM	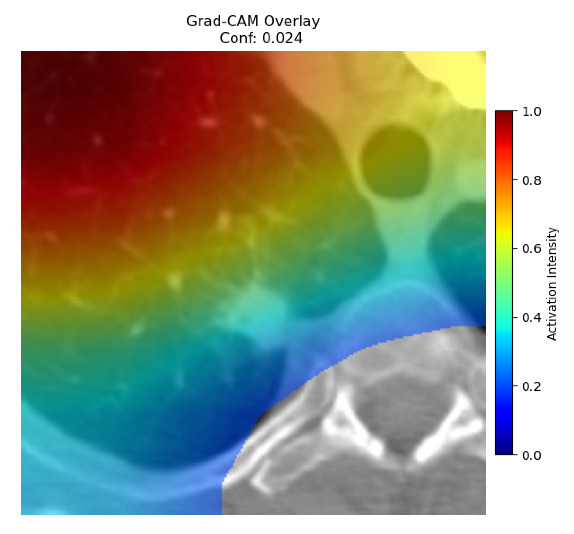	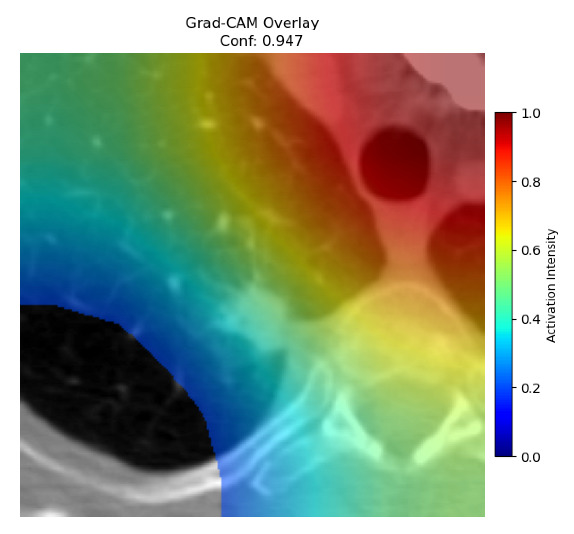	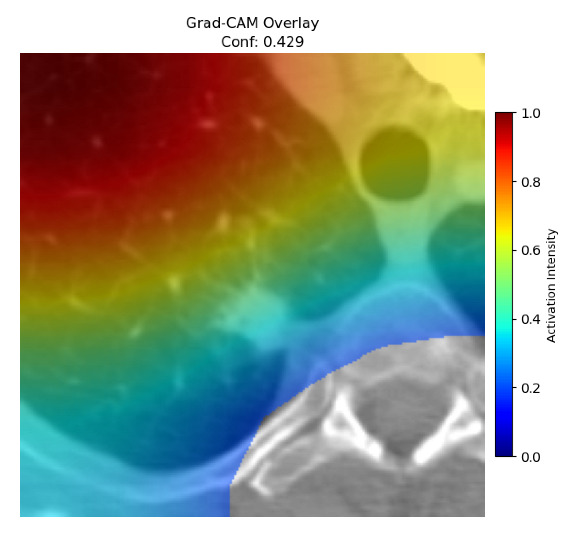	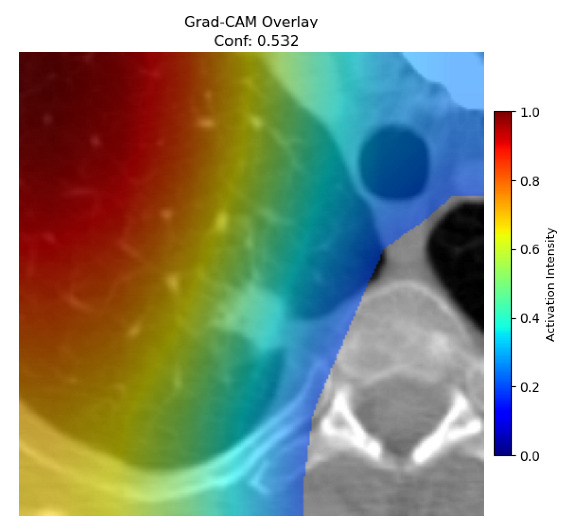
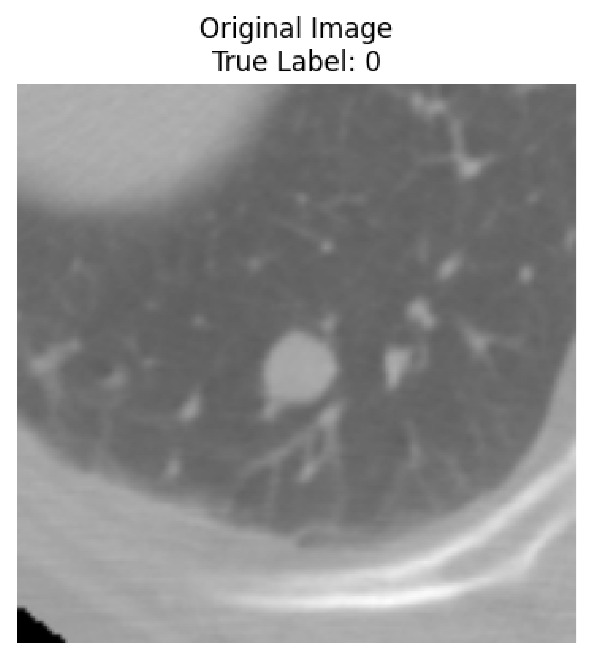	LIME	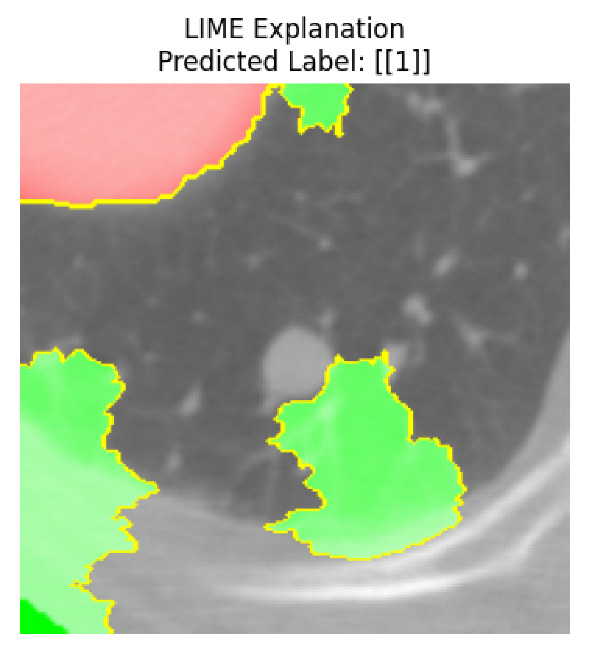	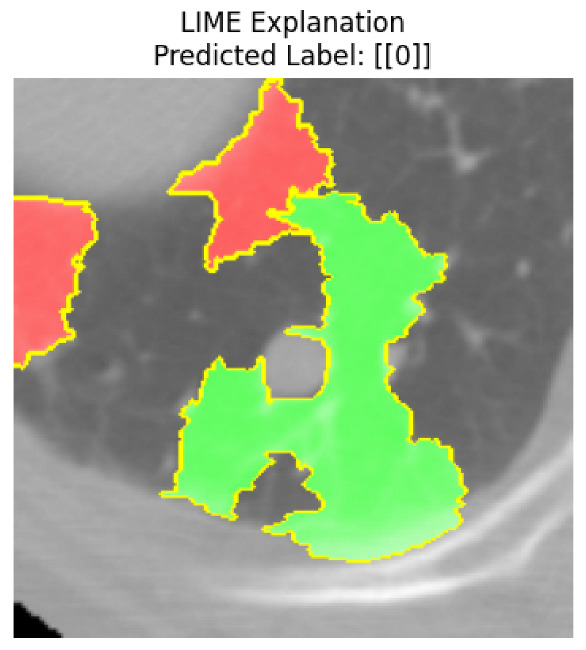	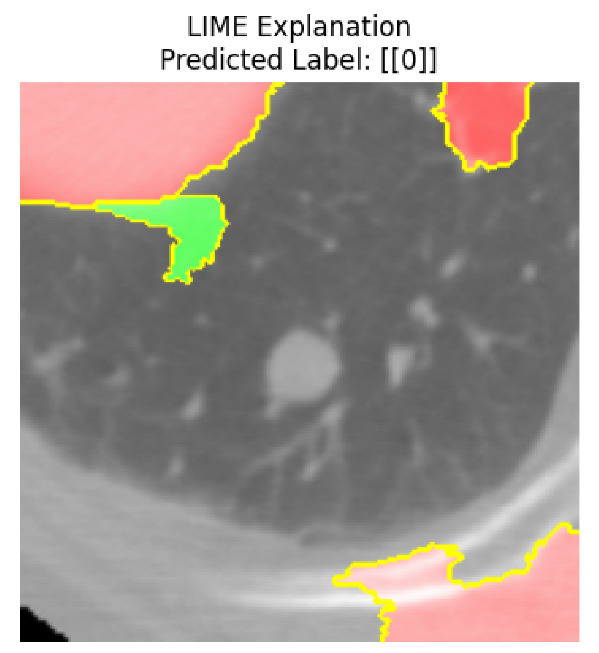	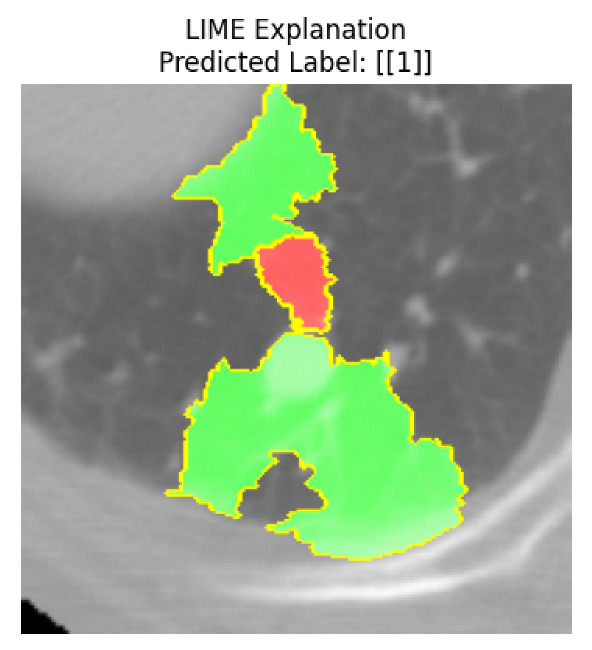
	Grad-CAM	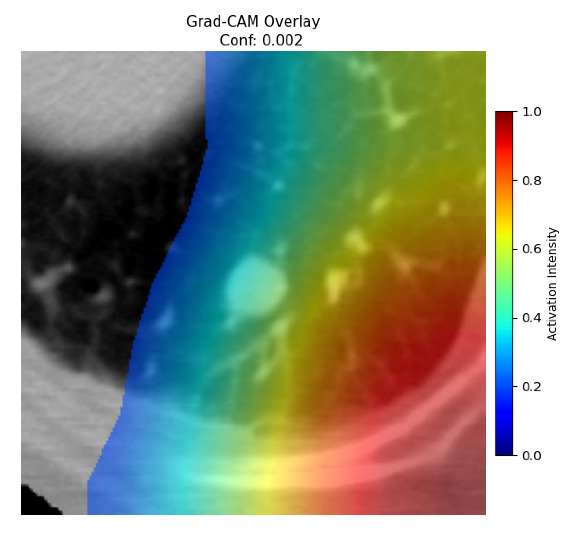	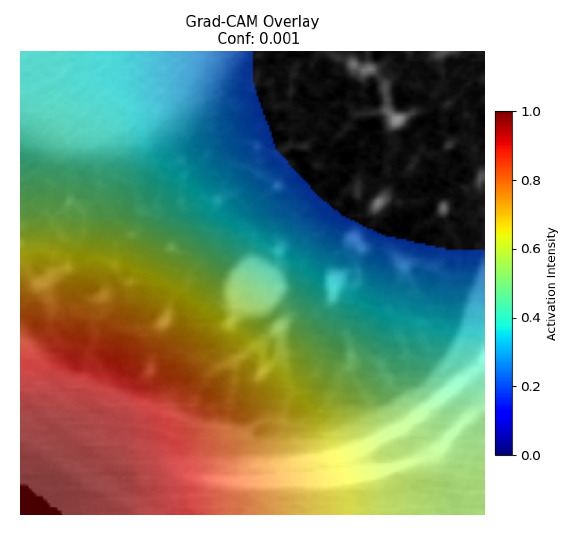	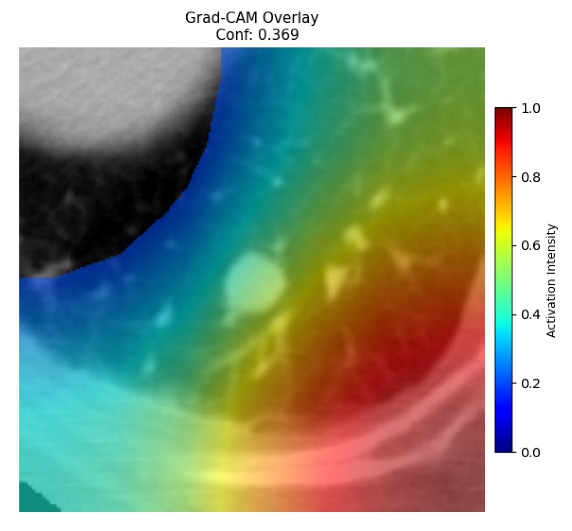	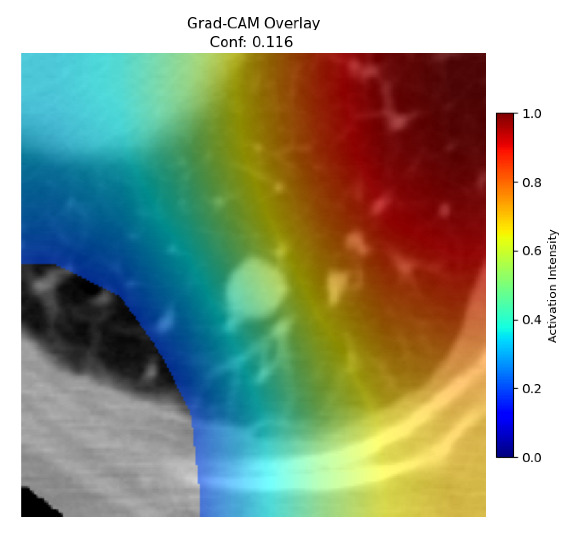
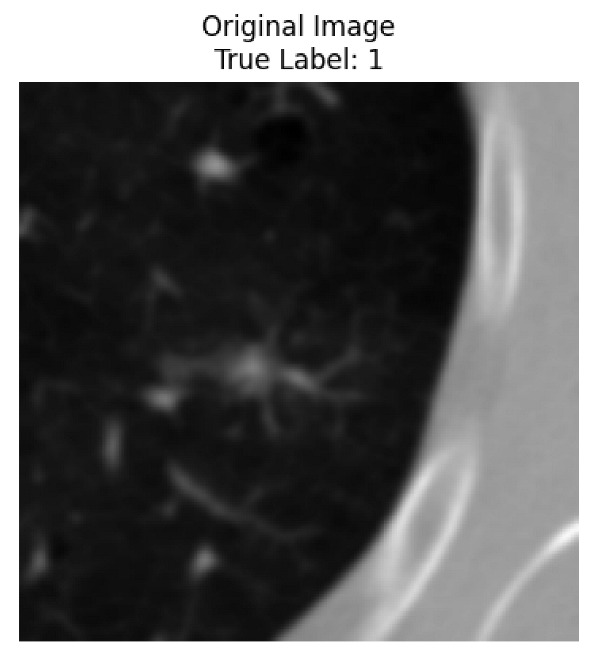	LIME	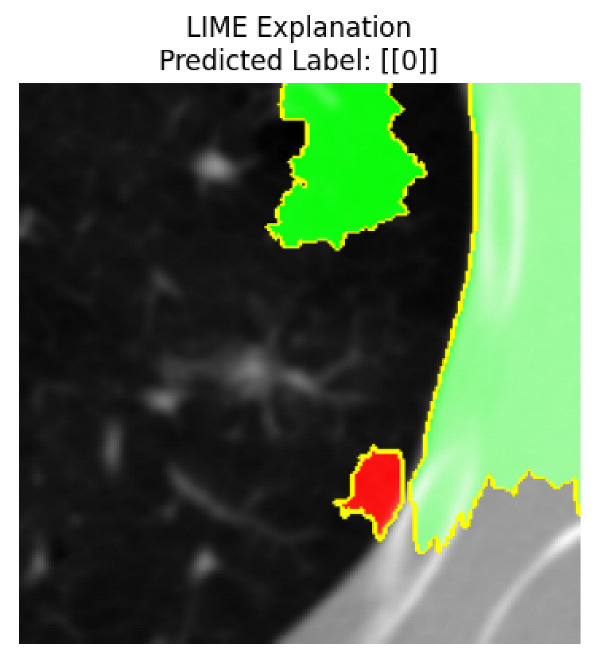	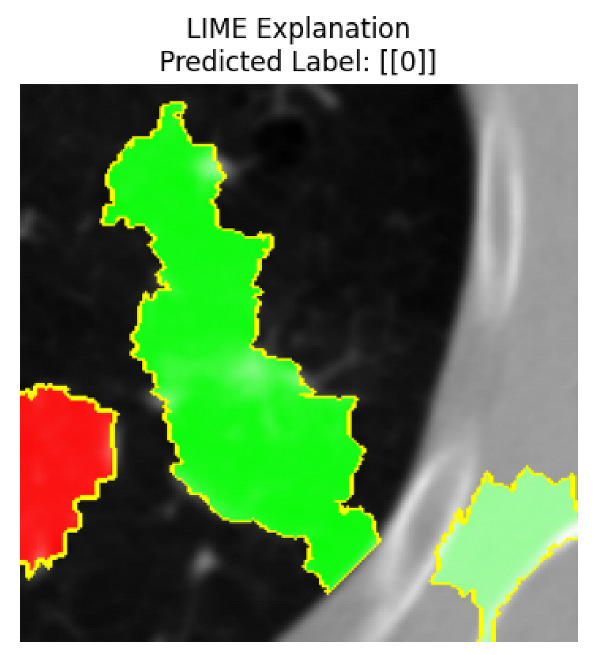	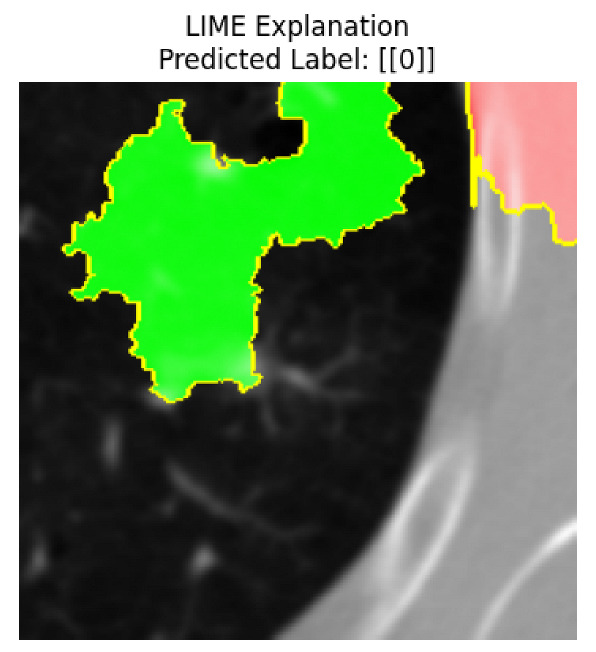	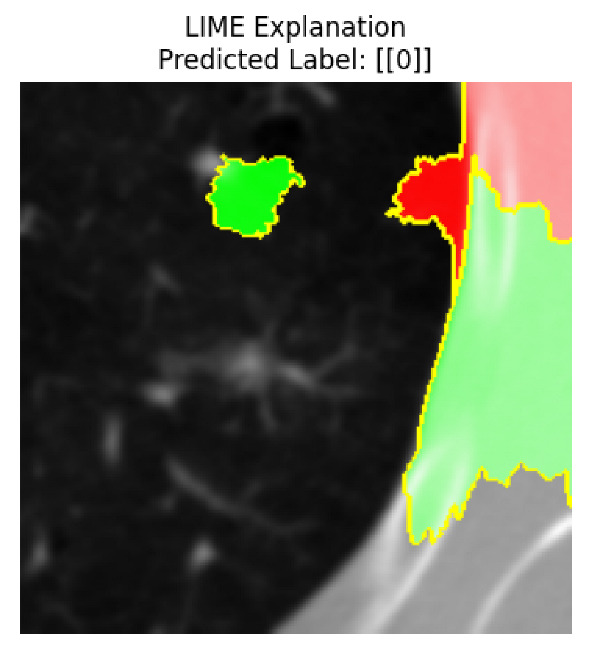
	Grad-CAM	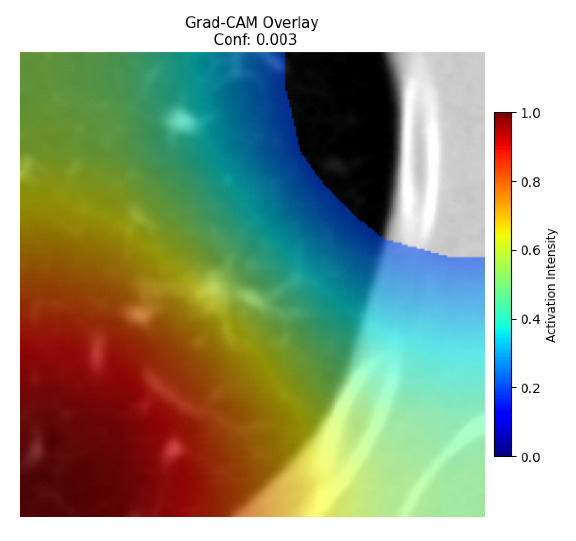	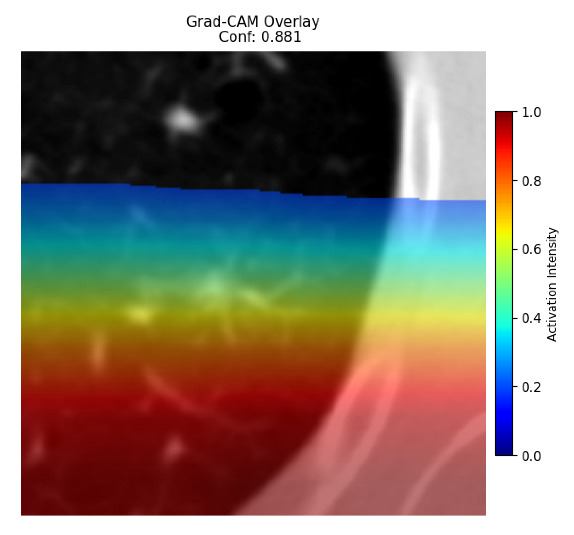	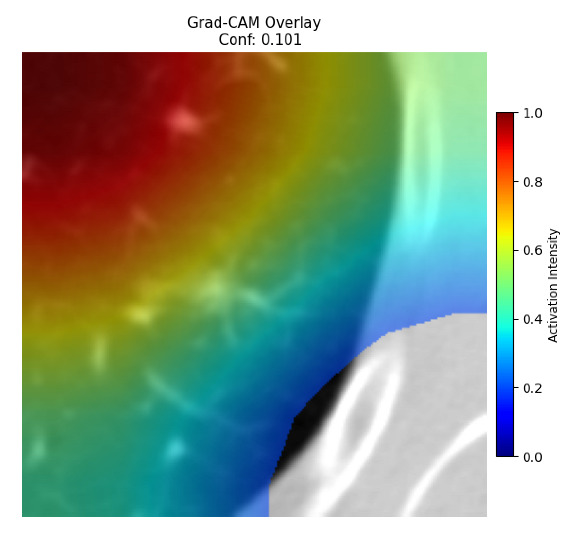	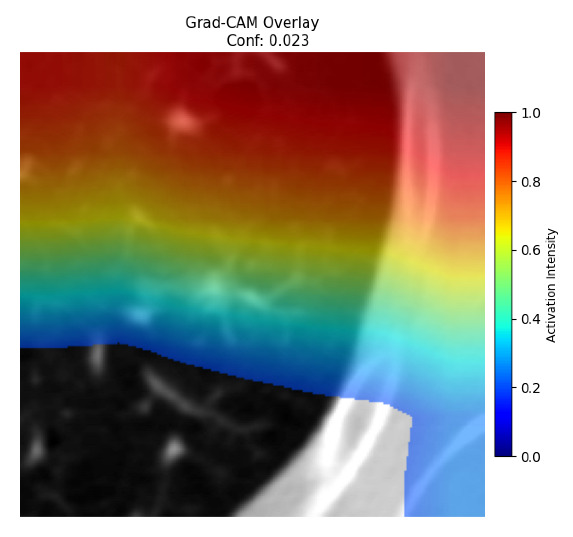
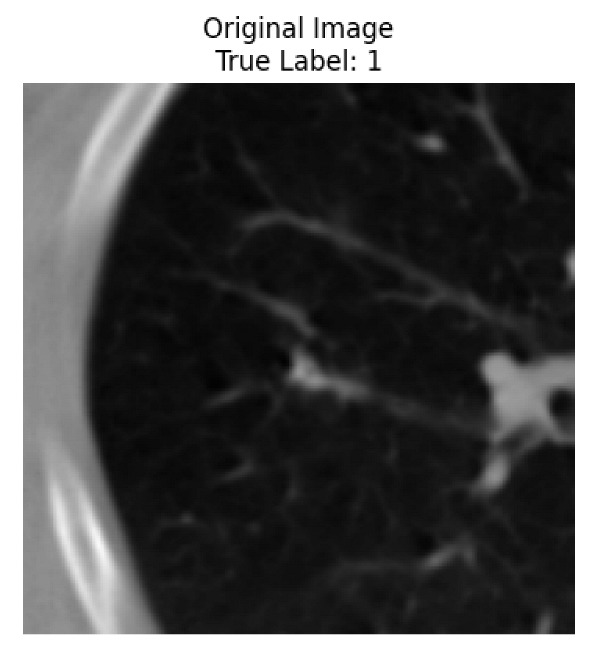	LIME	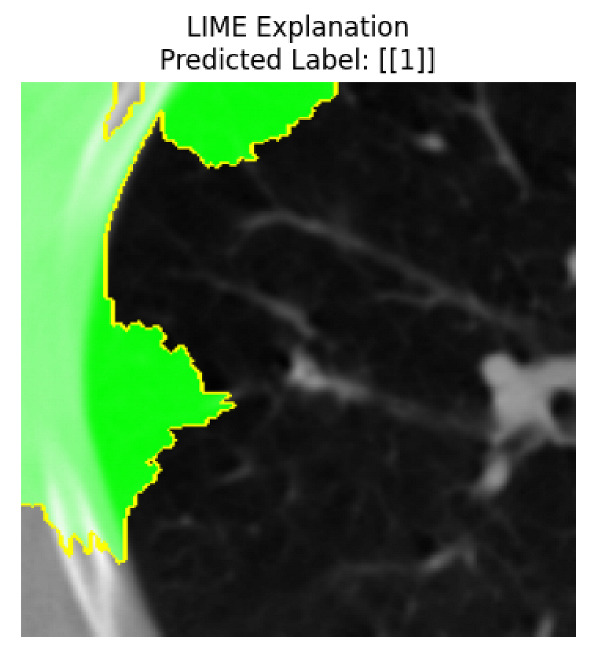	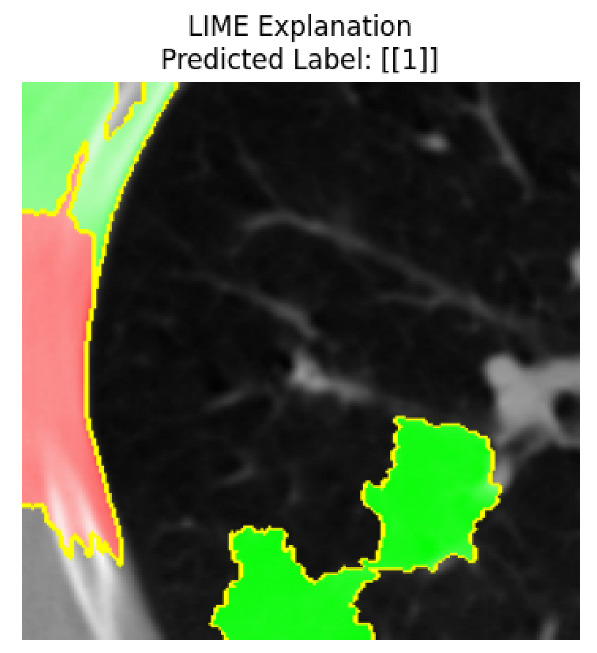	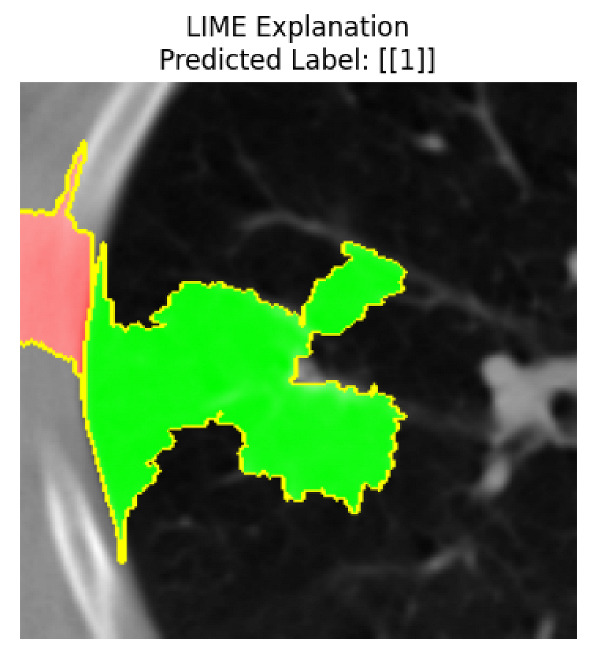	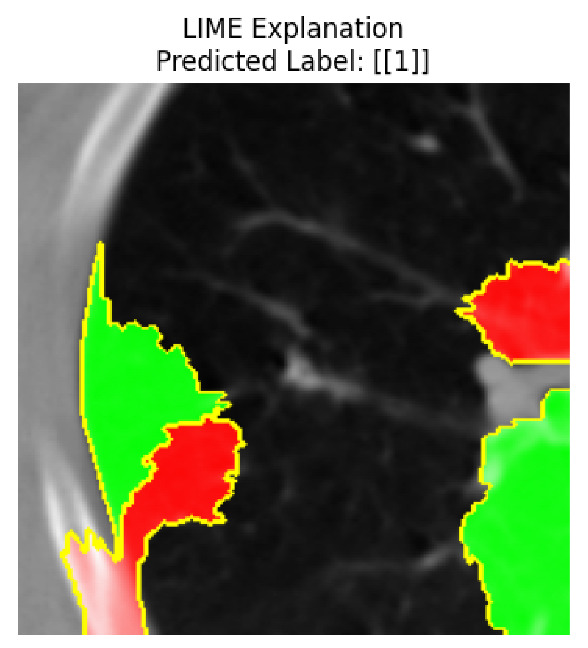
	Grad-CAM	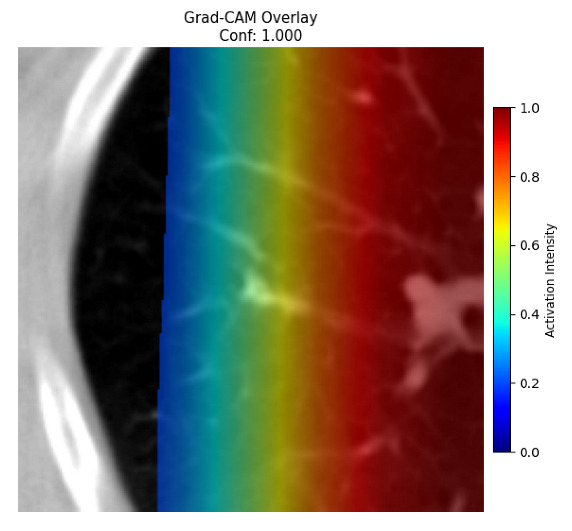	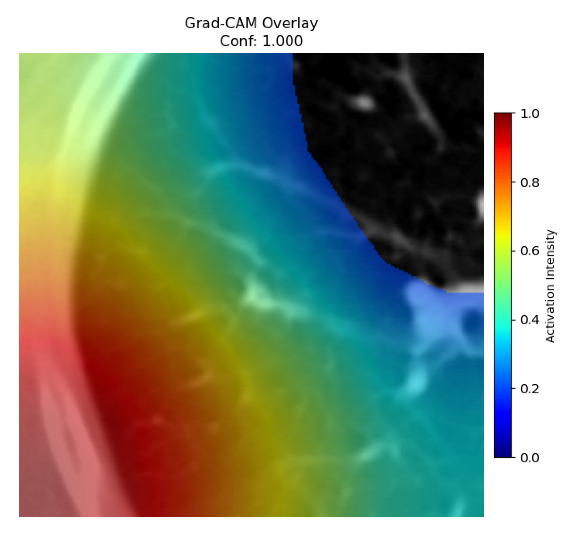	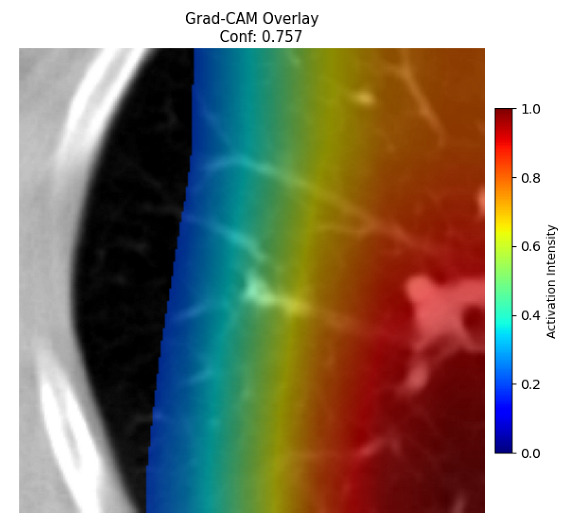	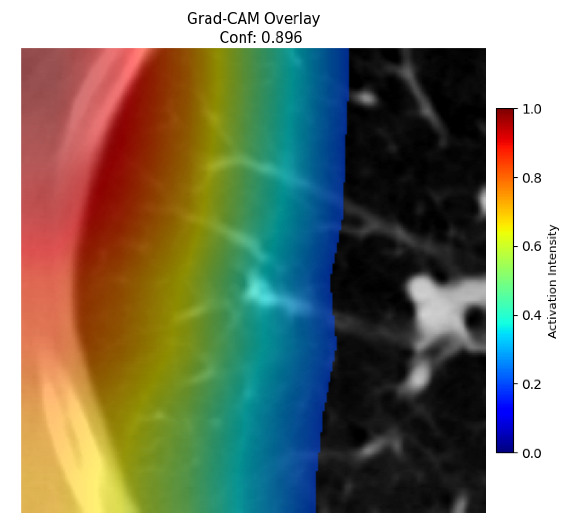

**Table 6:** LIME and Grad-CAM explanations generated by four lightweight CNN variants using **EfficientNetB0** as backbone, applied to four representative CT images.

Original Image&True Label	XAI	2D-CNN Attention	2D-CNN Without Att	1D-CNN Attention	1D-CNN Without Att
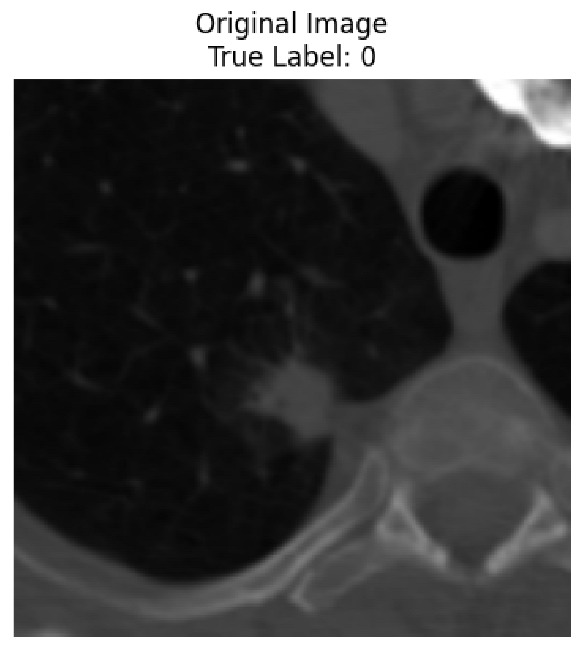	LIME	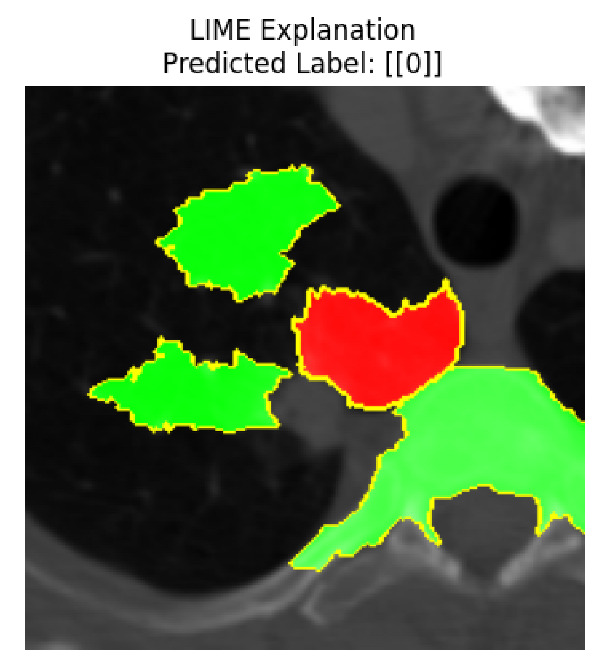	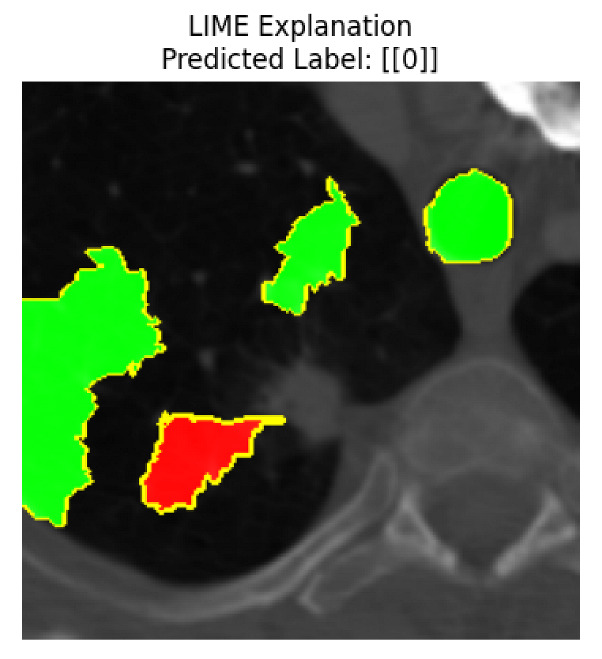	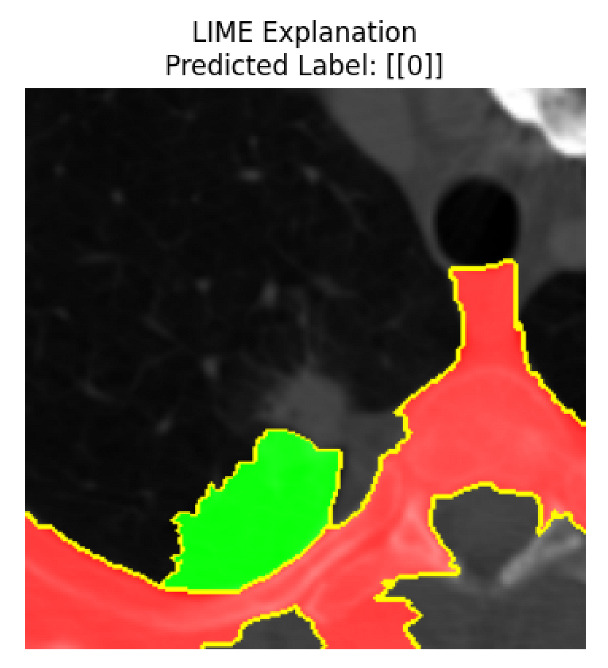	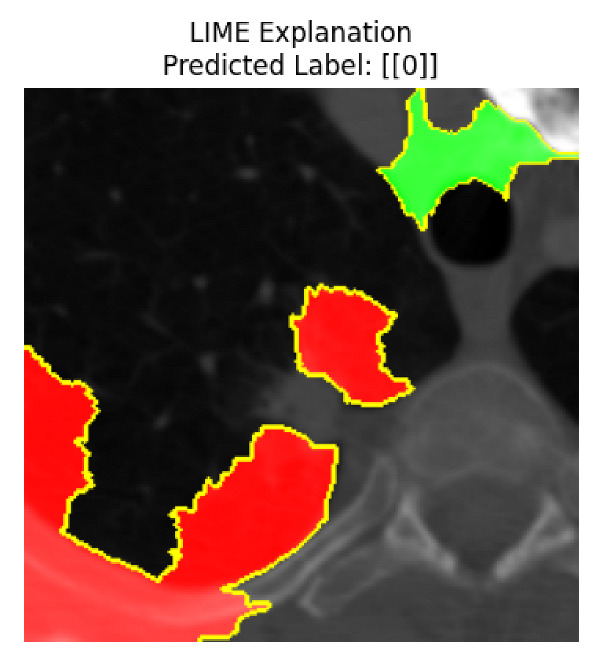
	Grad-CAM	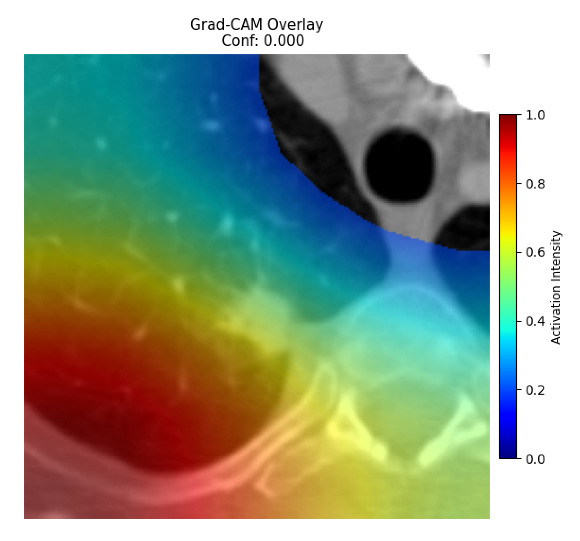	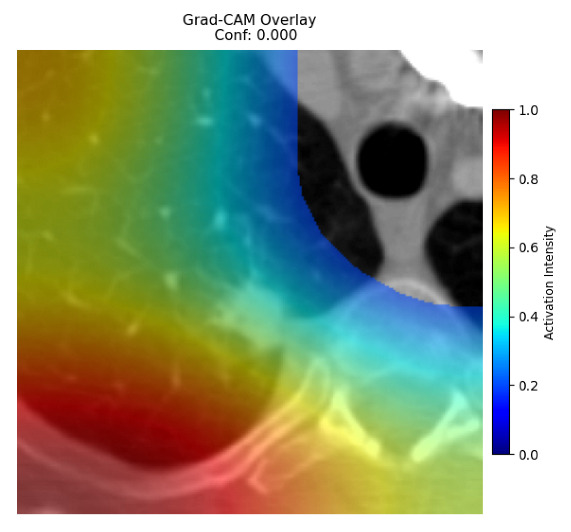	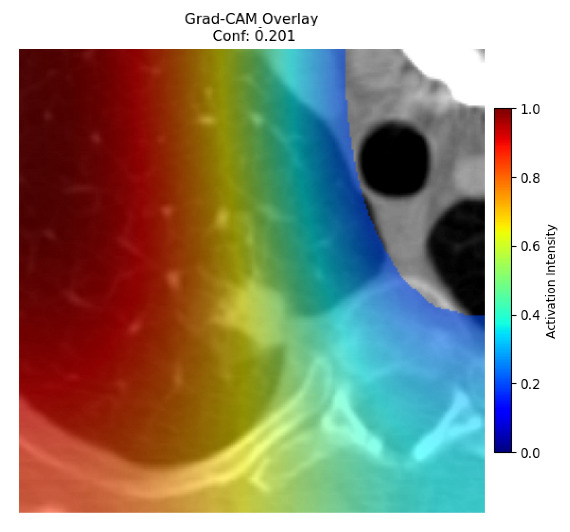	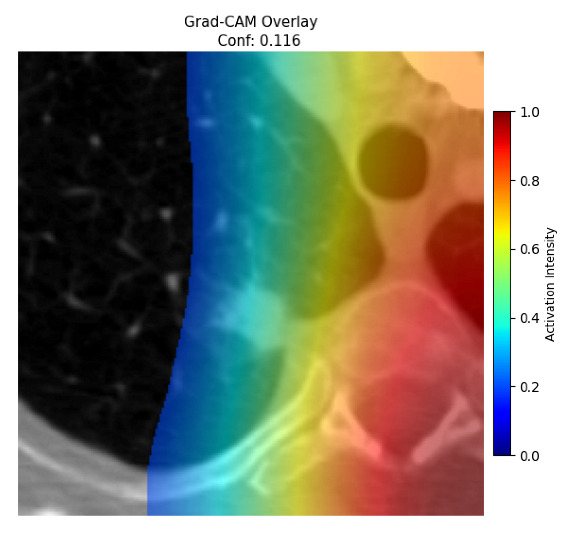
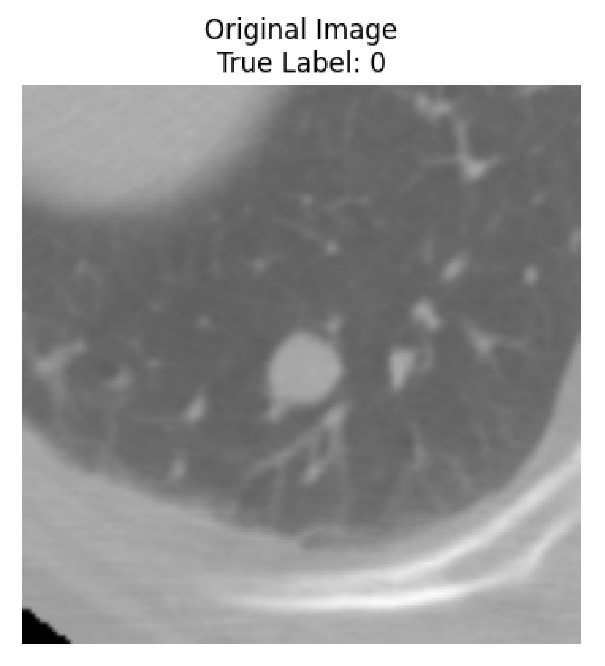	LIME	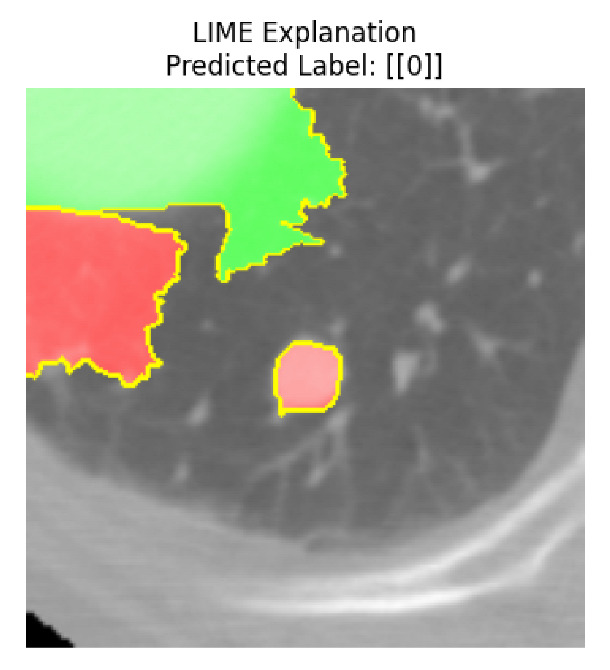	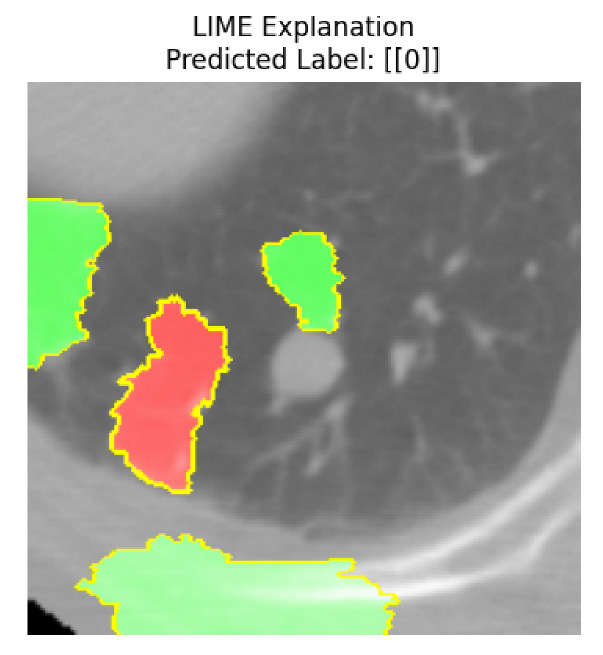	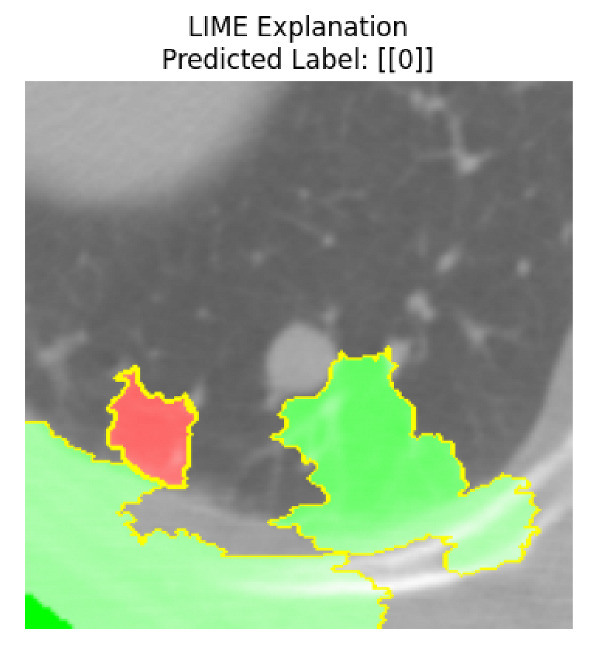	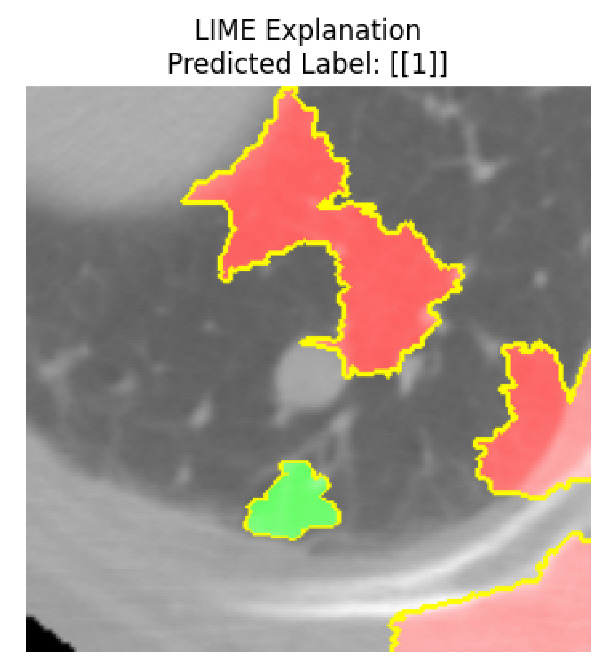
	Grad-CAM	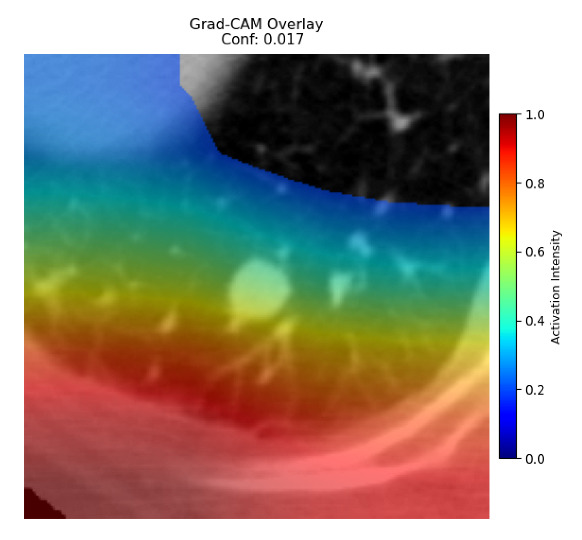	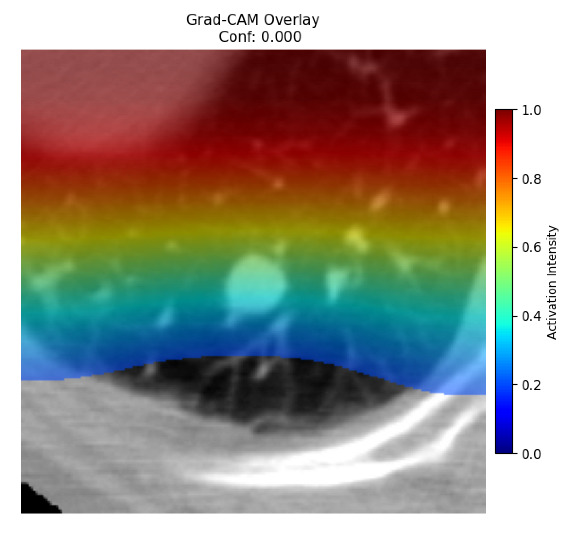	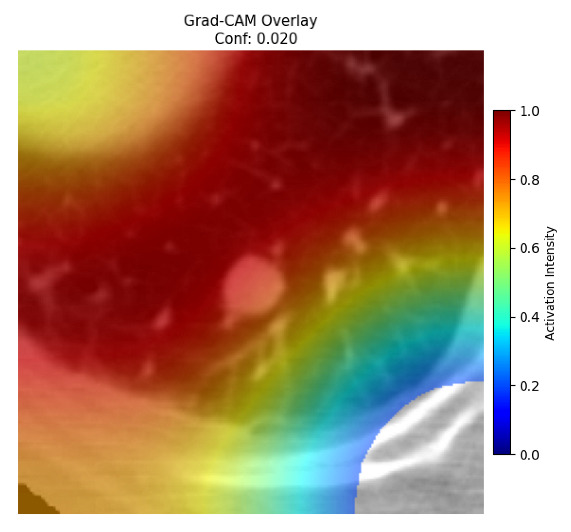	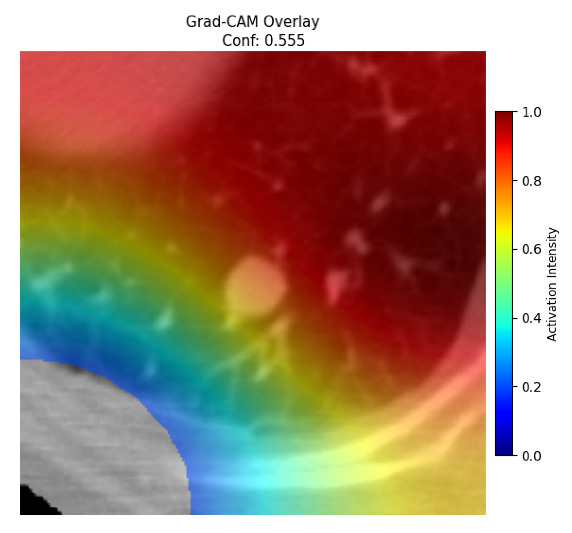
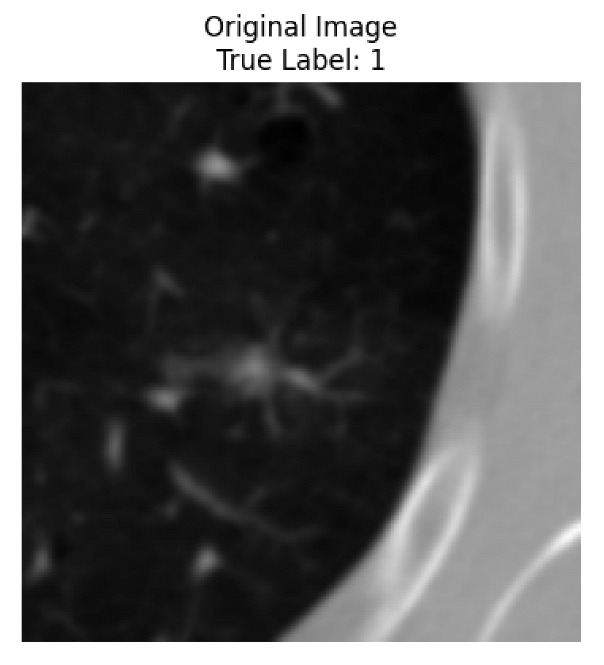	LIME	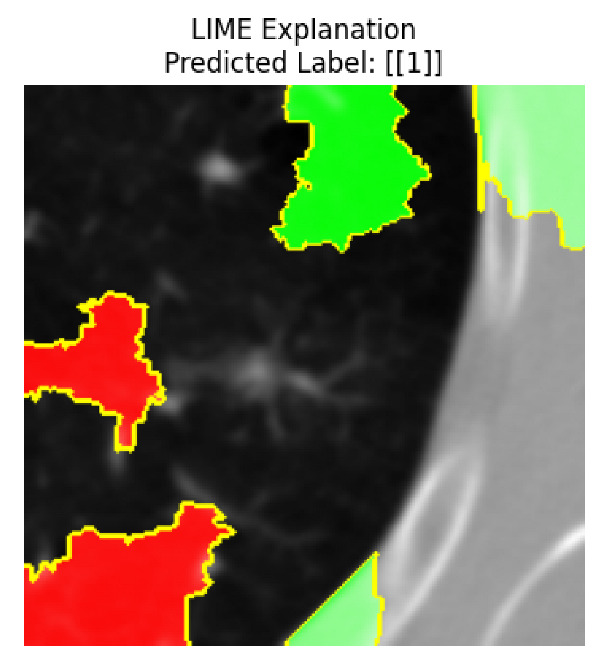	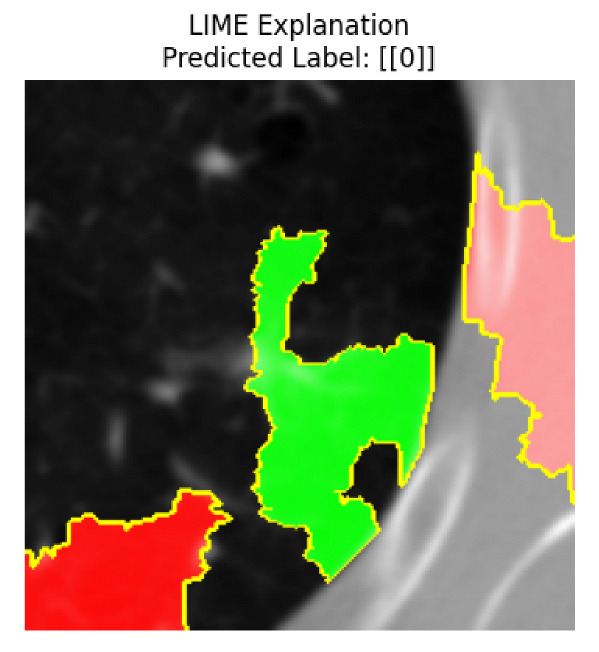	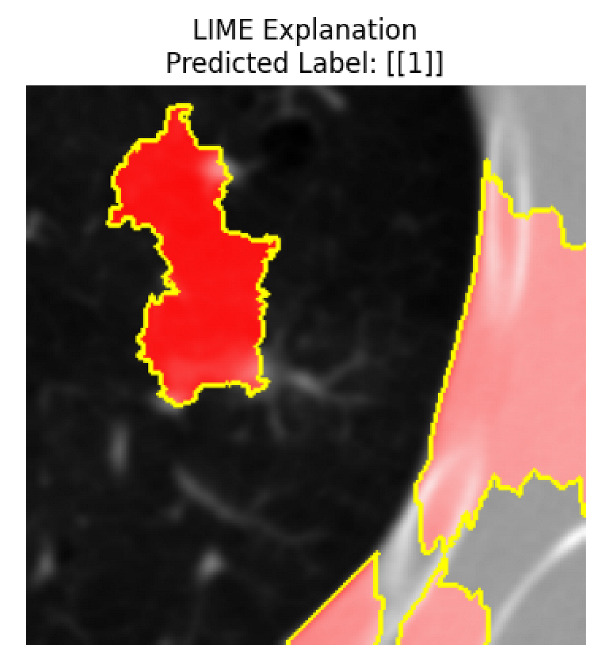	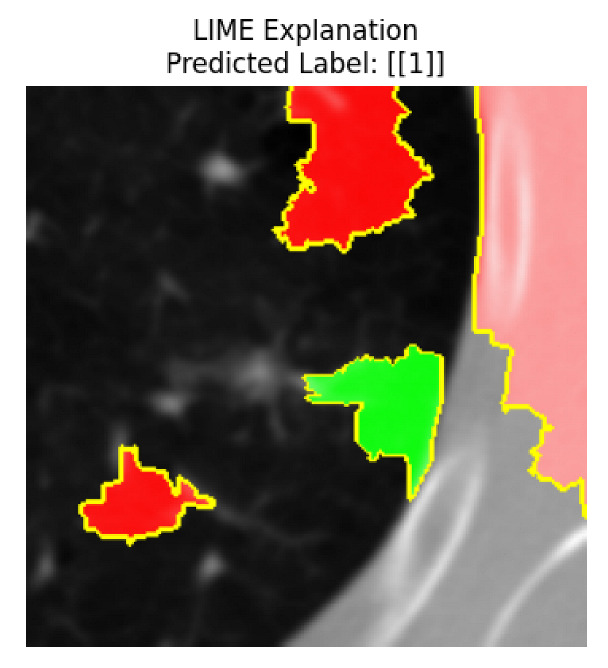
	Grad-CAM	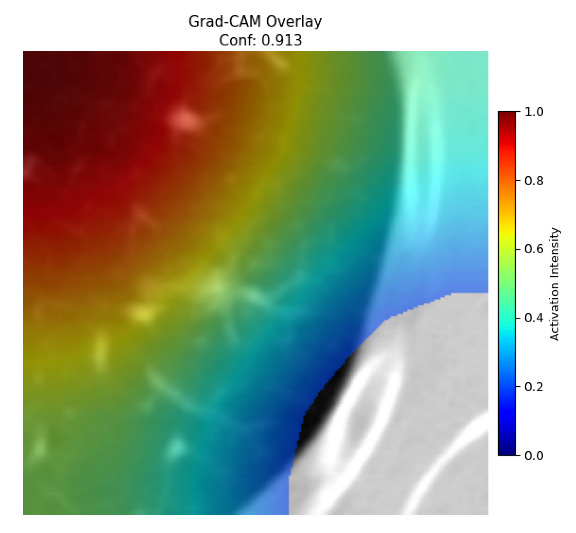	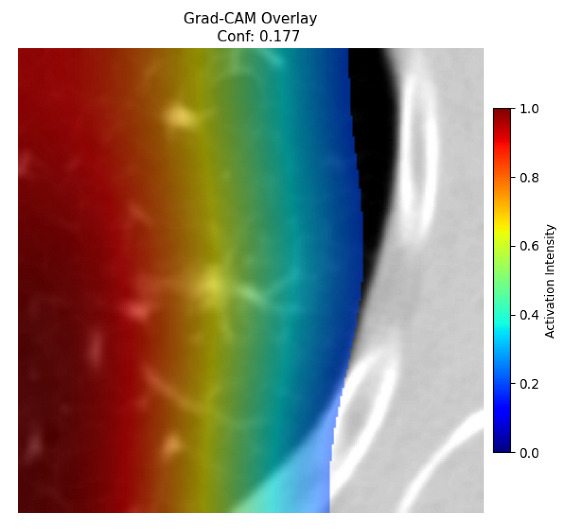	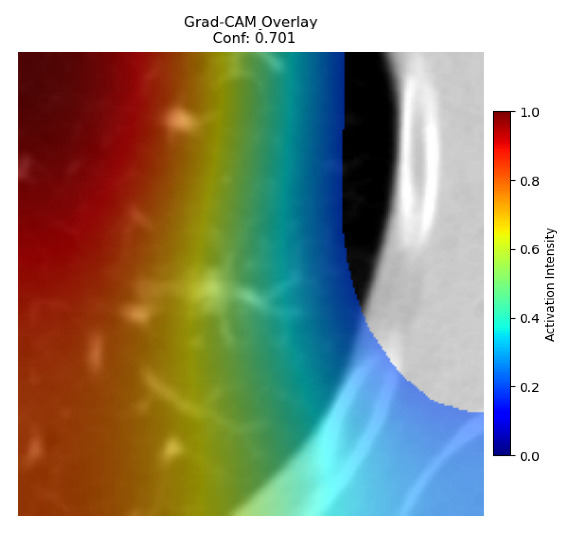	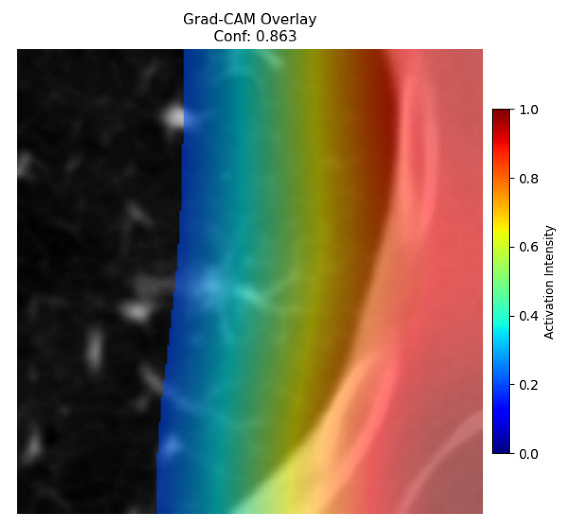
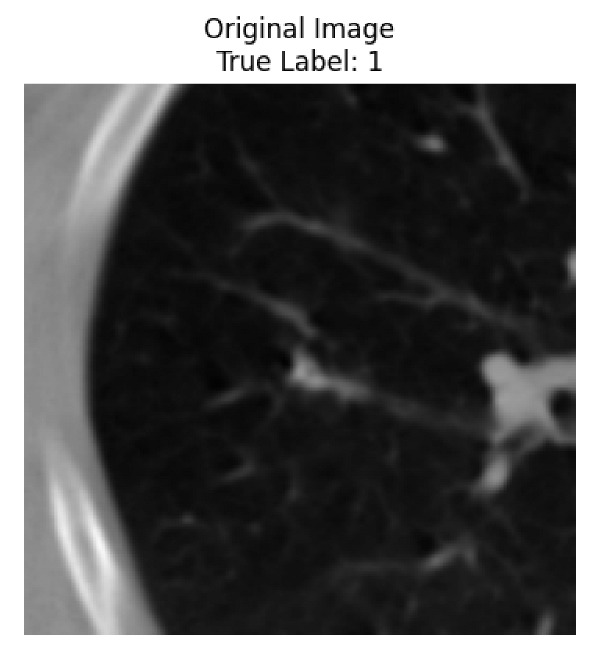	LIME	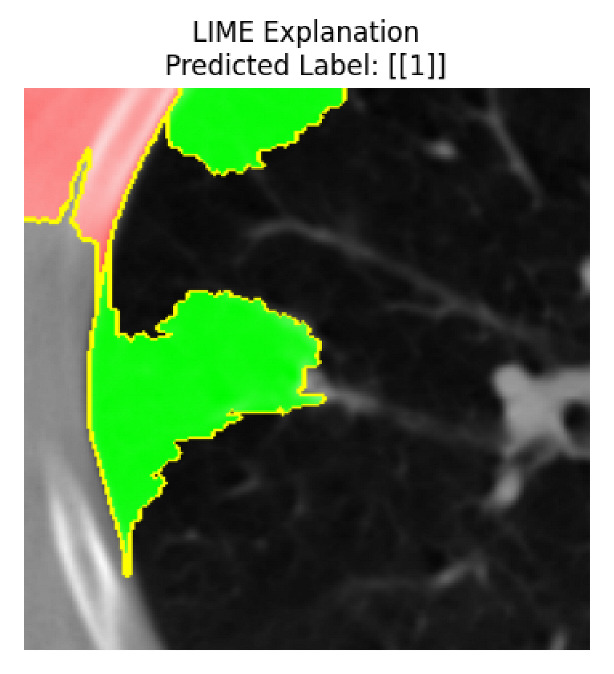	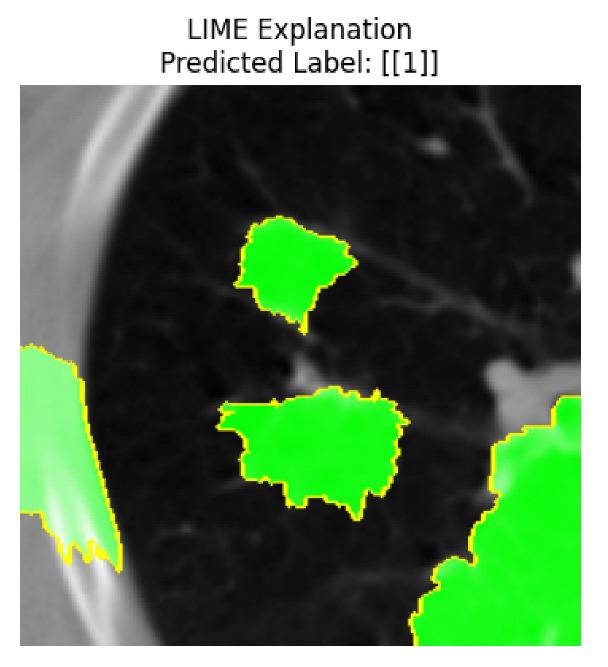	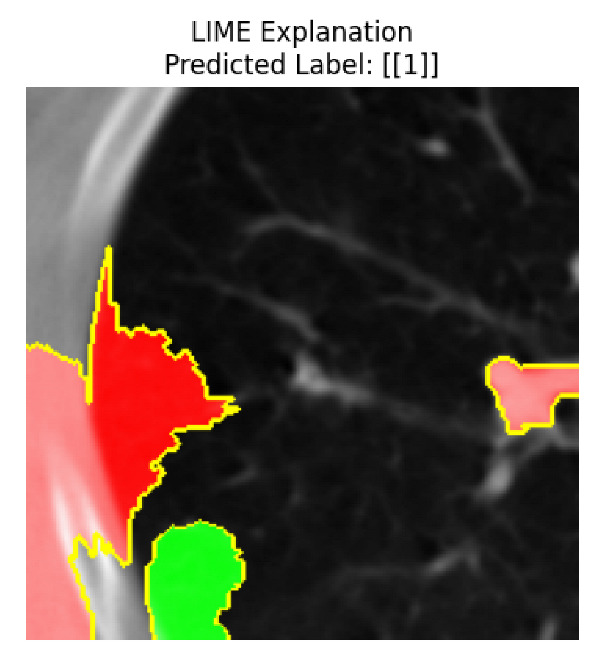	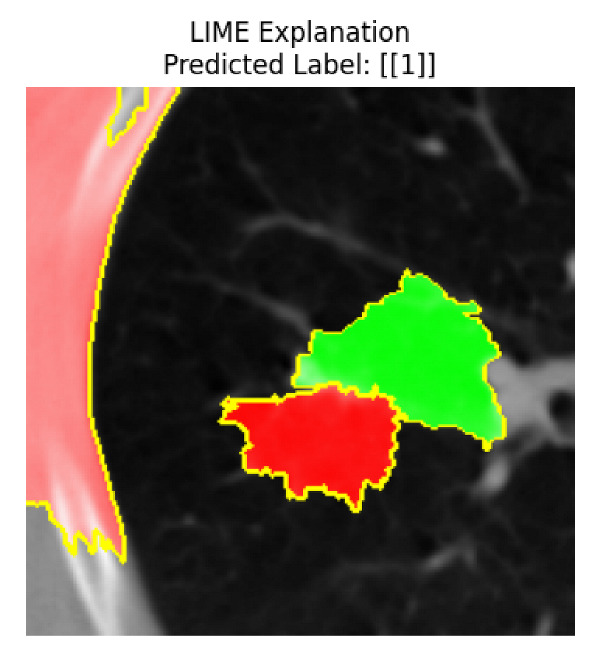
	Grad-CAM	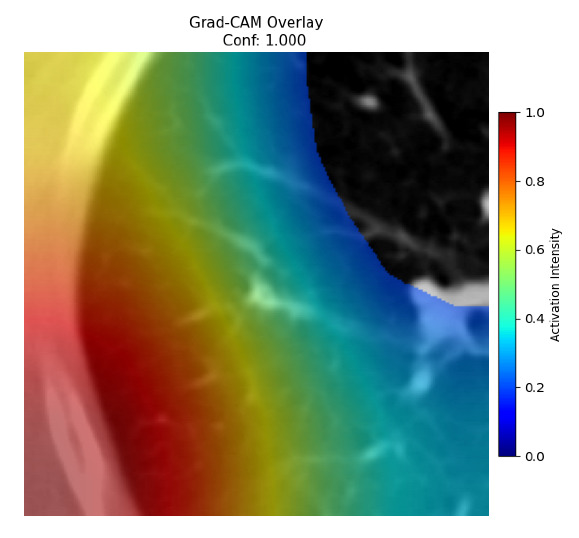	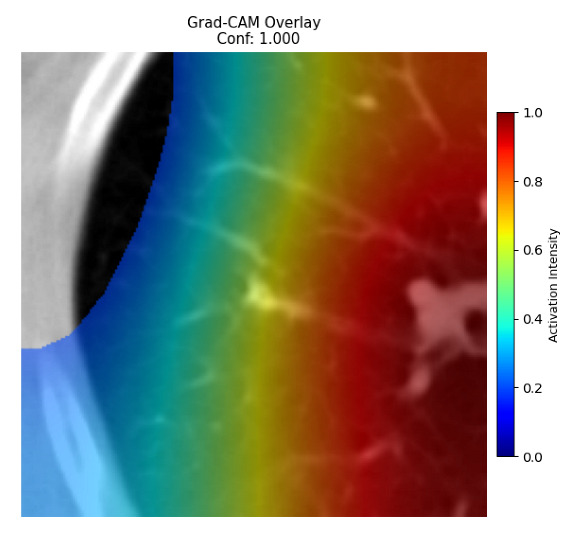	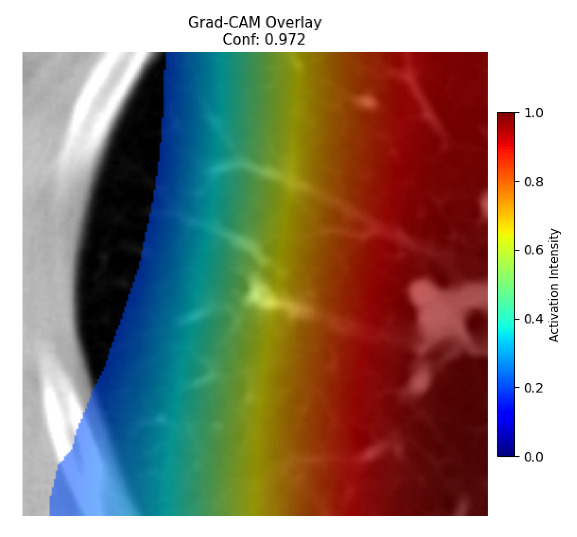	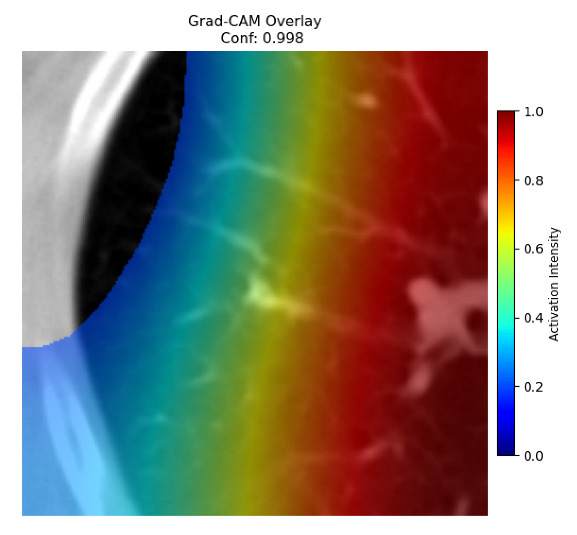

**Table 7:** LIME and Grad-CAM explanations generated by four lightweight CNN variants using **ConvNeXt-Tiny** as backbone, applied to four representative CT images.

Original Image&True Label	XAI	2D-CNN Attention	2D-CNN Without Att	1D-CNN Attention	1D-CNN Without Att
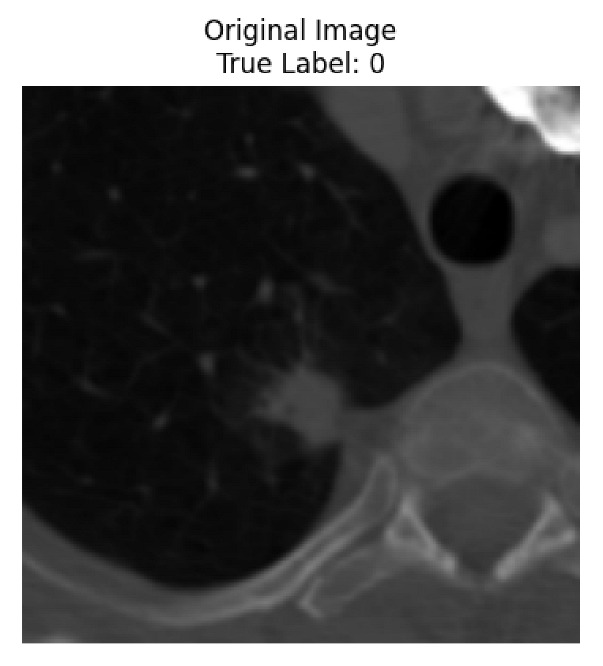	LIME	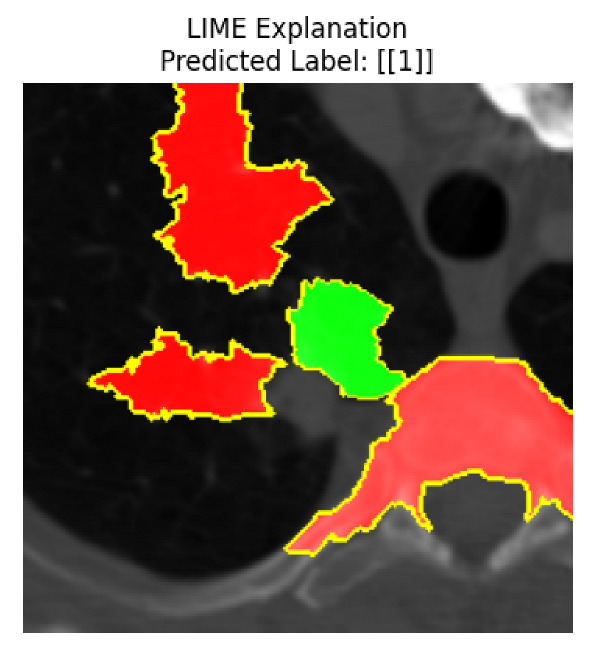	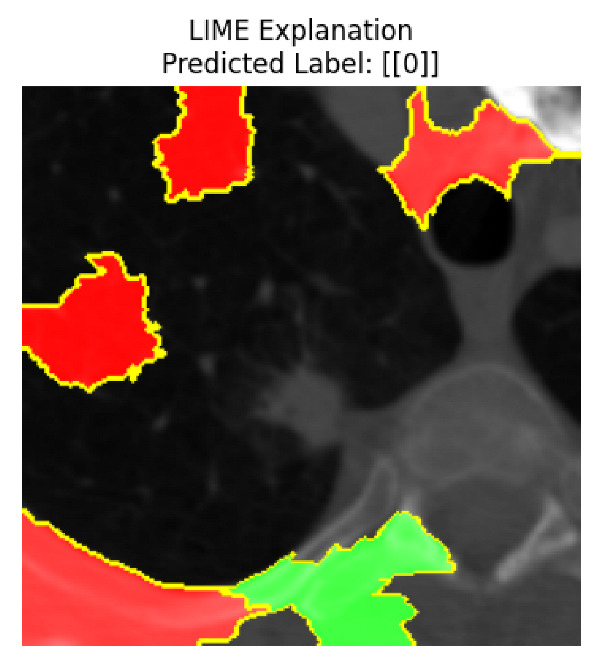	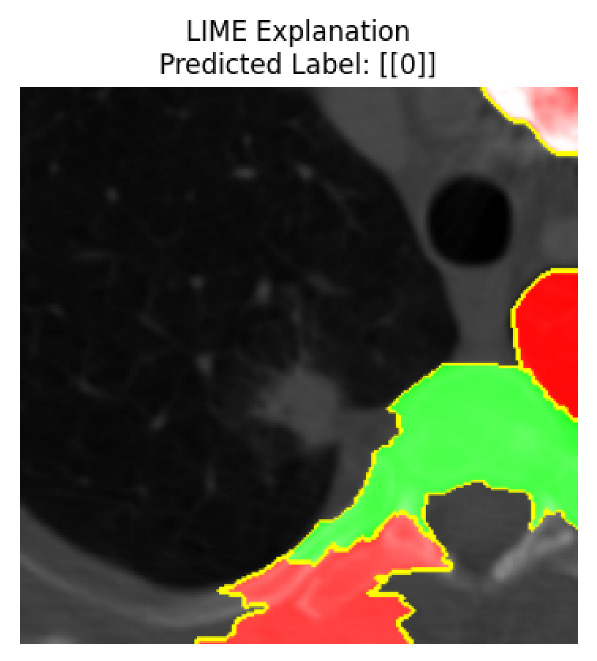	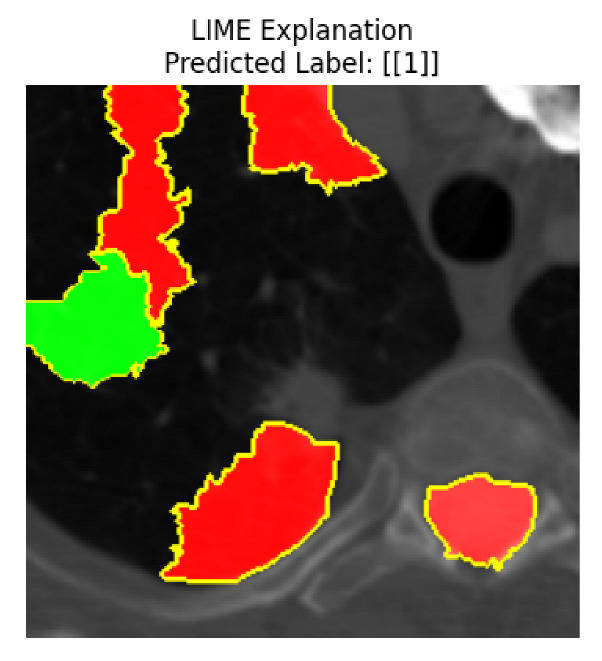
	Grad-CAM	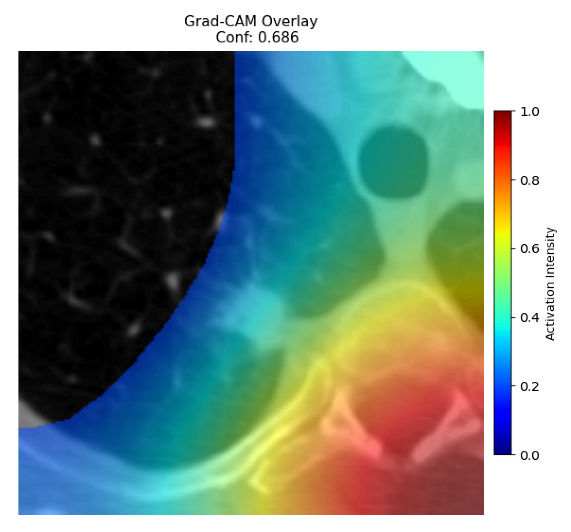	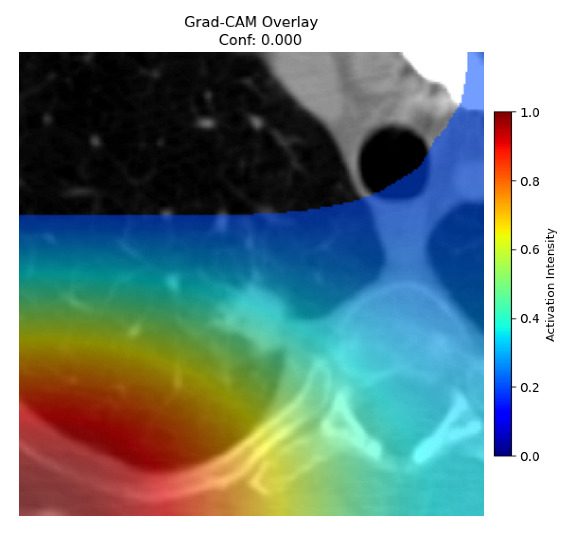	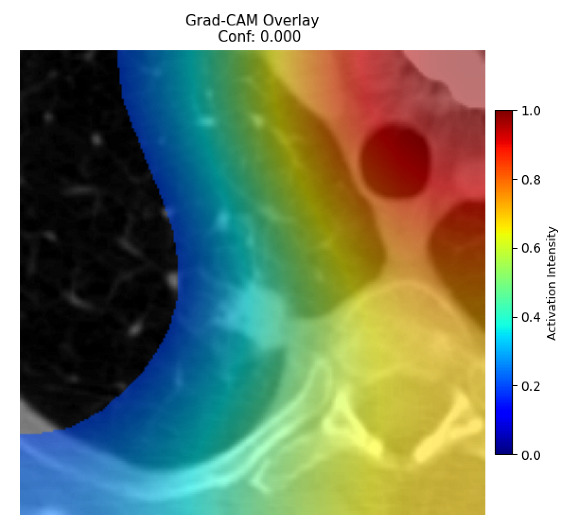	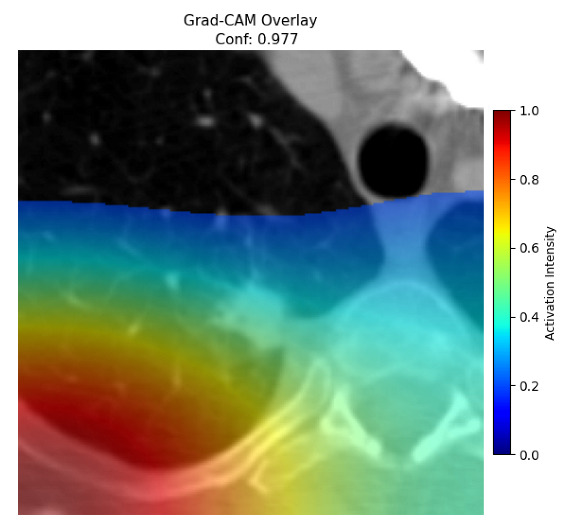
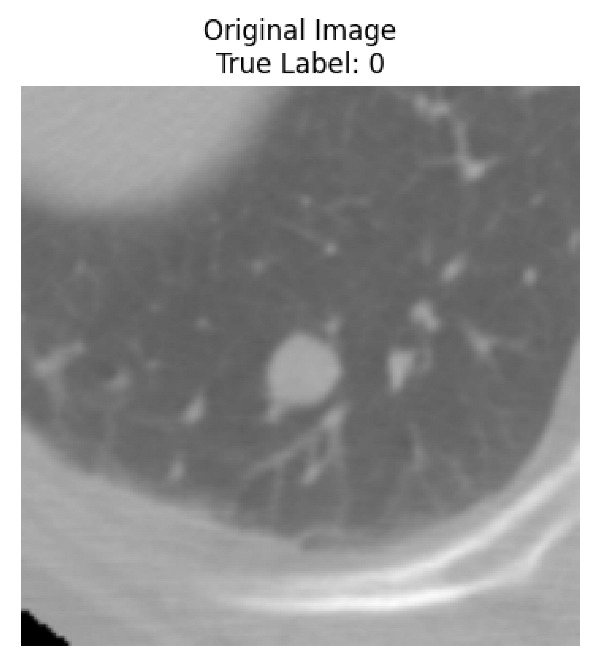	LIME	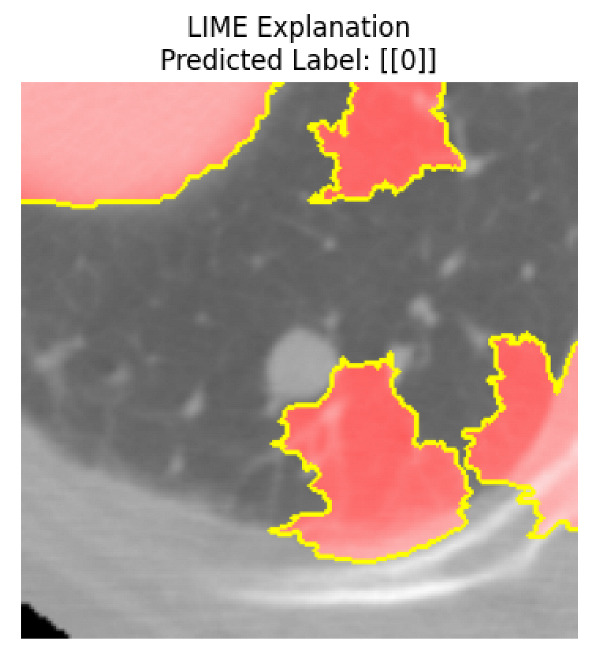	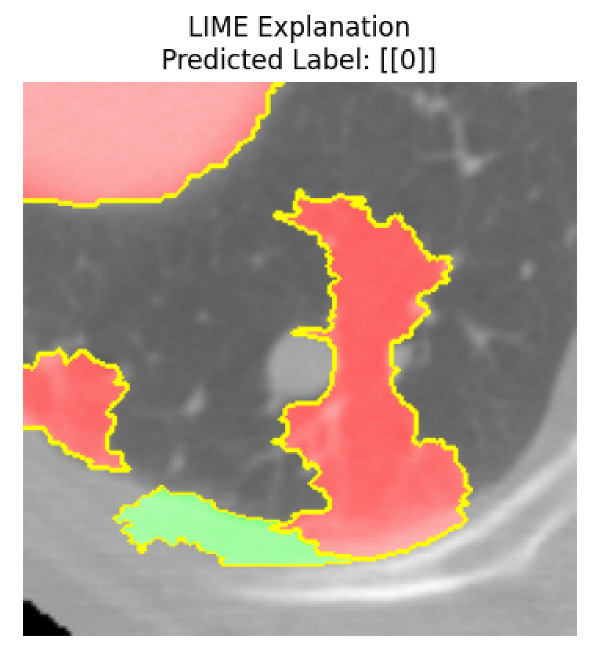	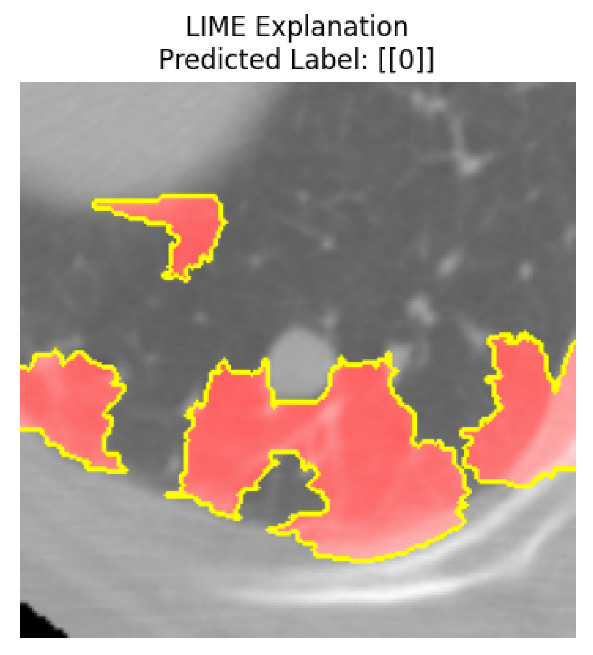	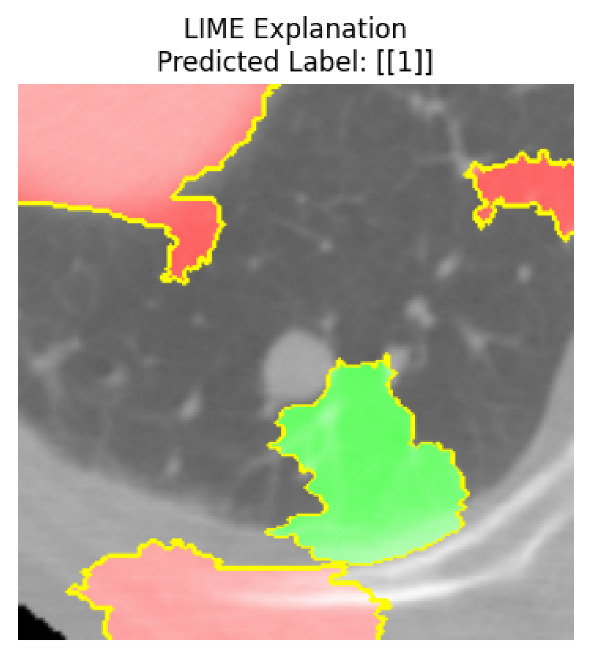
	Grad-CAM	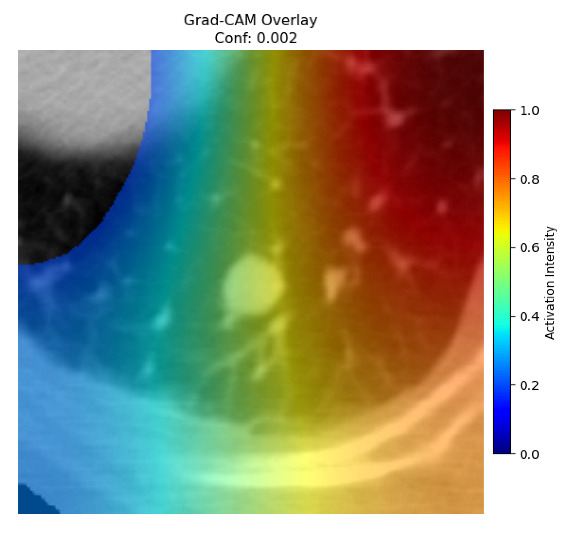	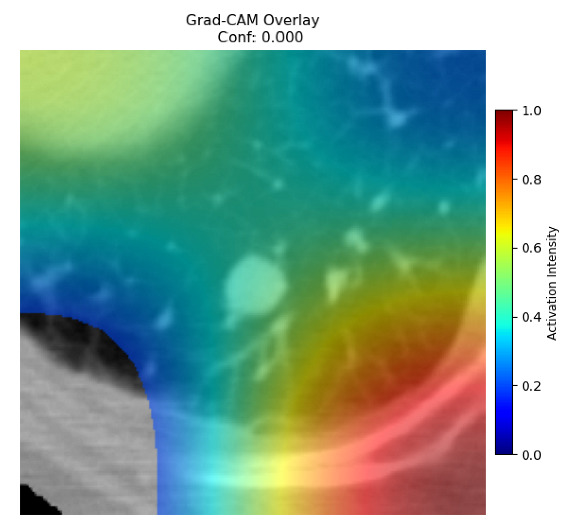	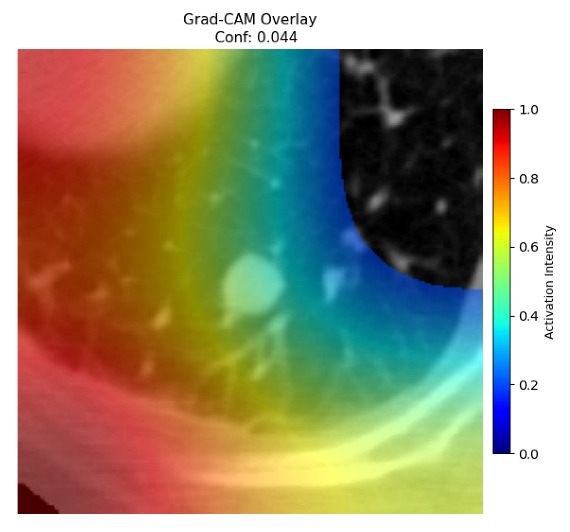	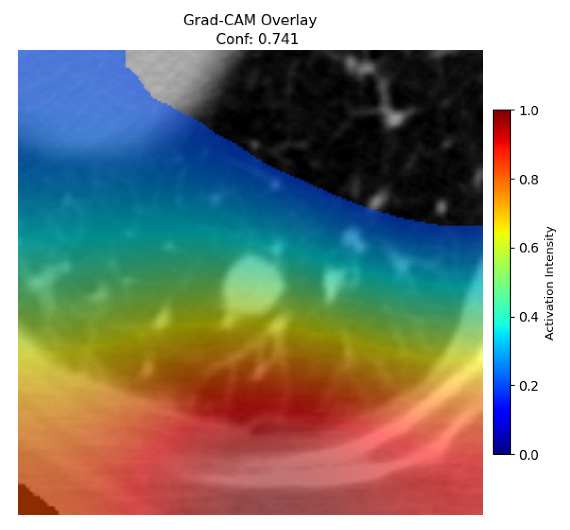
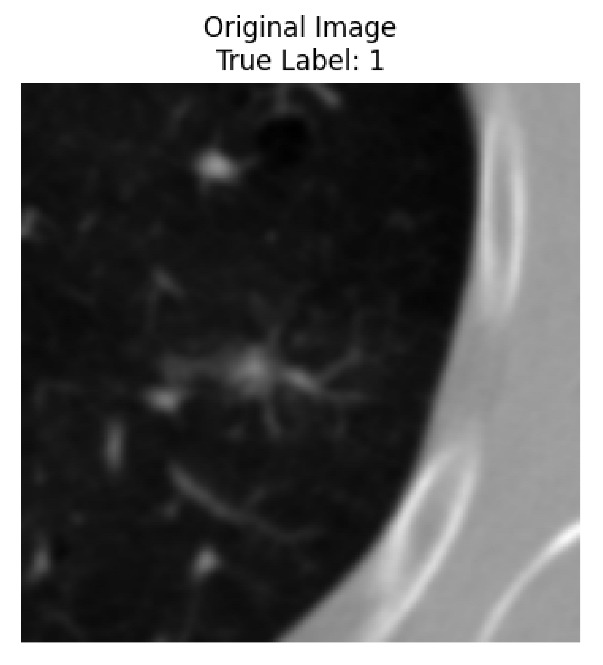	LIME	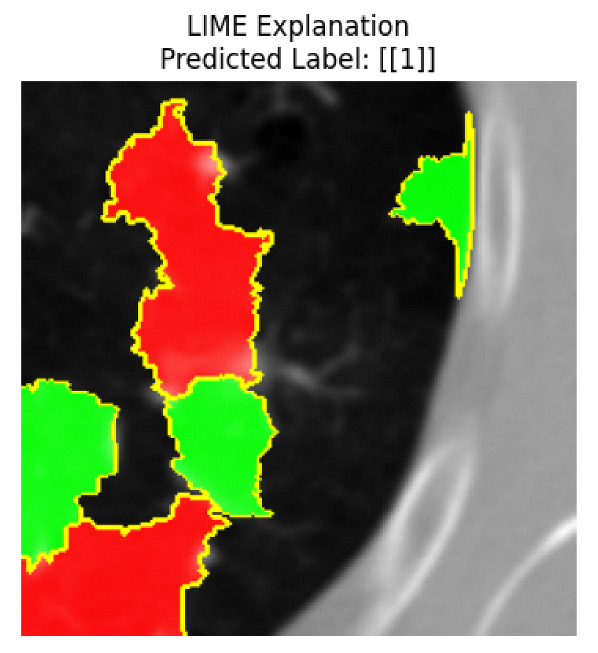	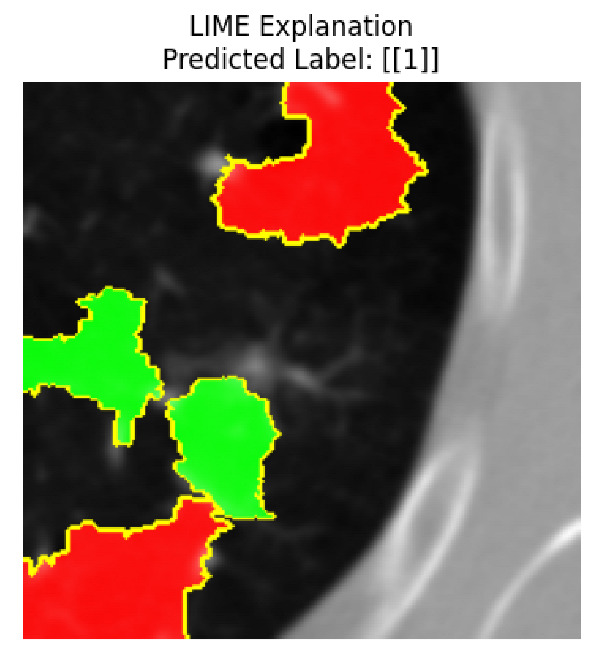	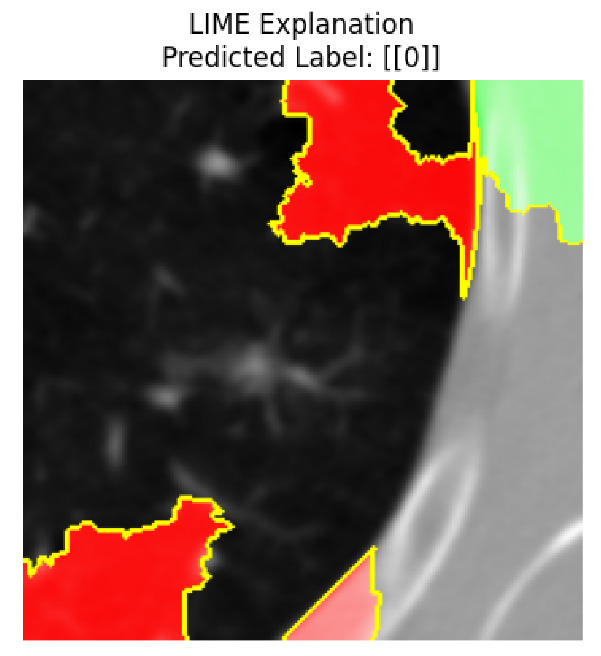	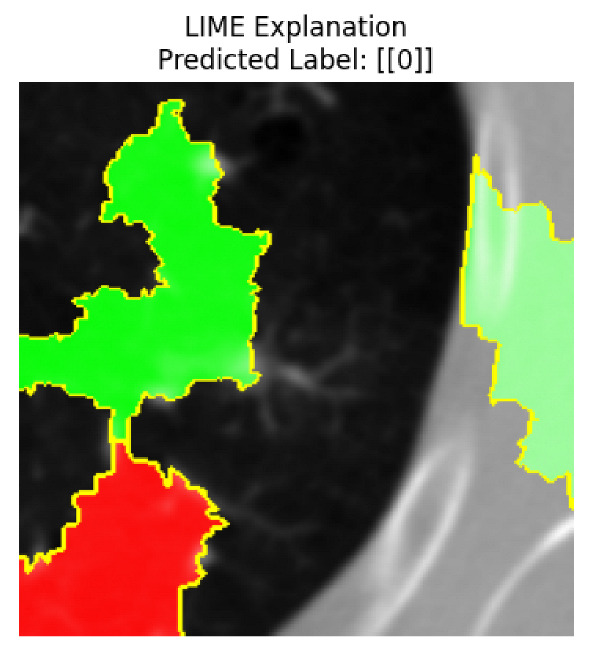
	Grad-CAM	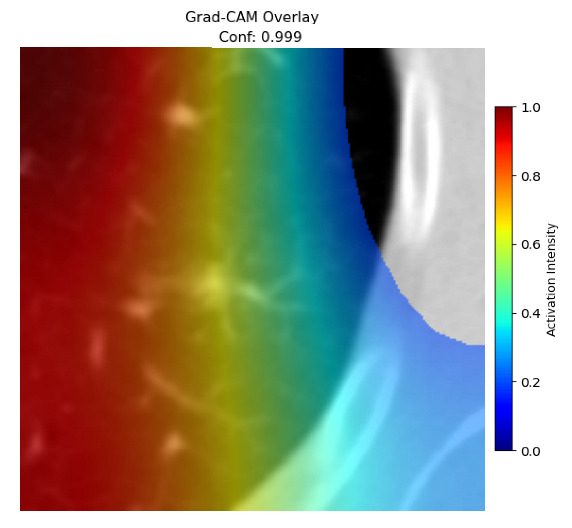	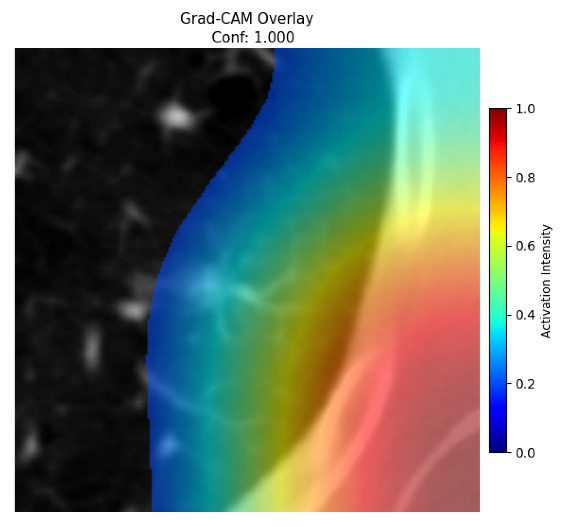	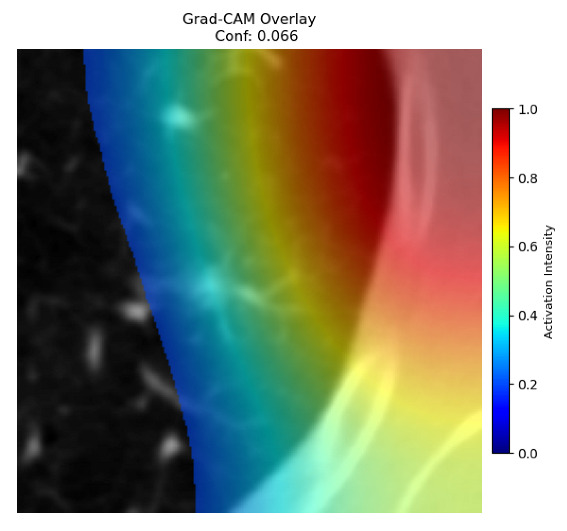	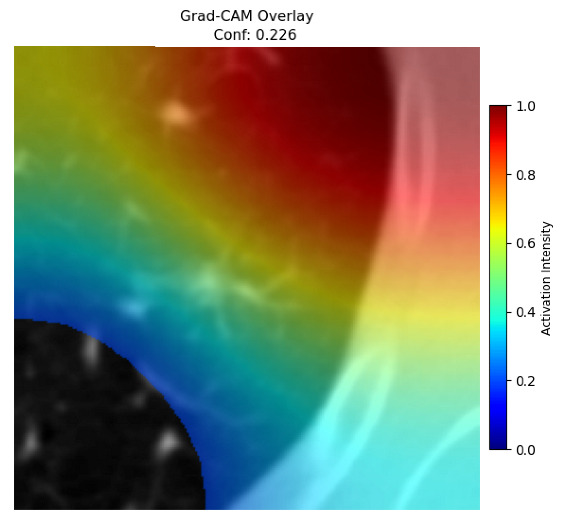
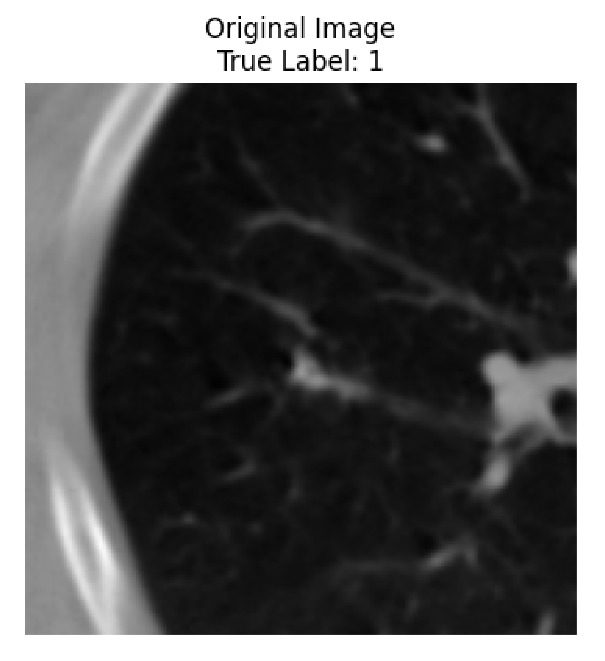	LIME	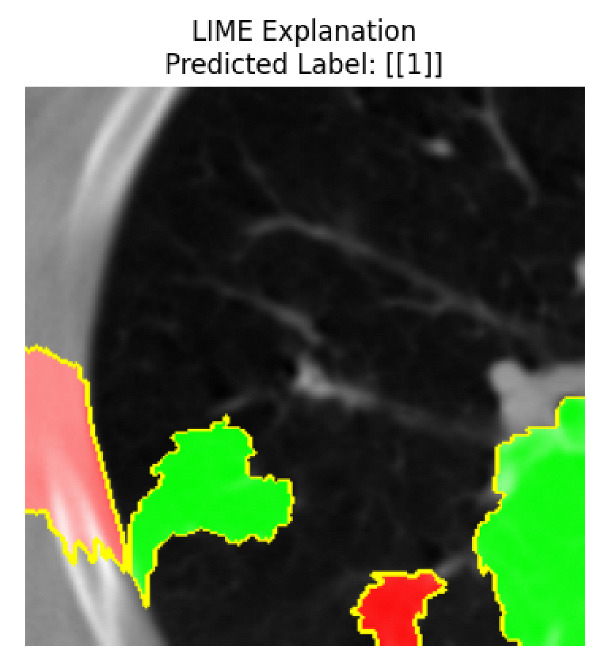	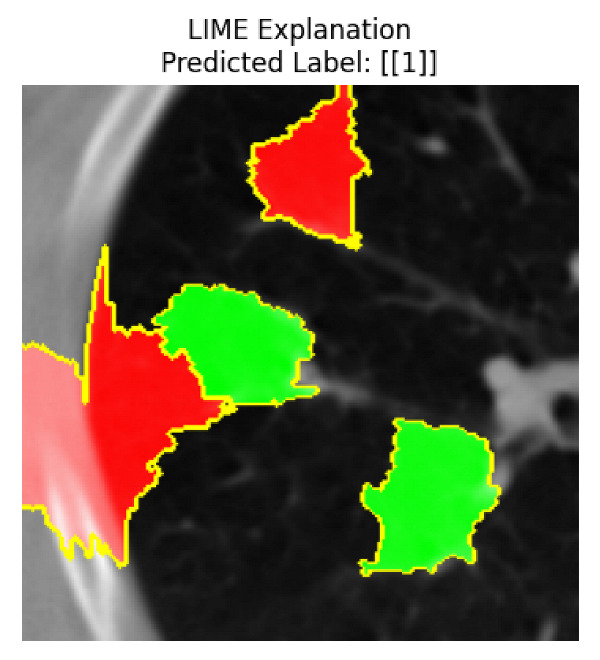	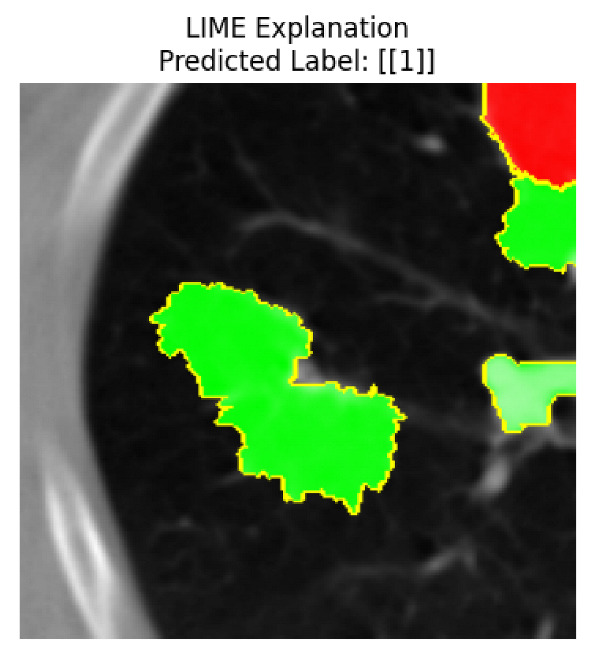	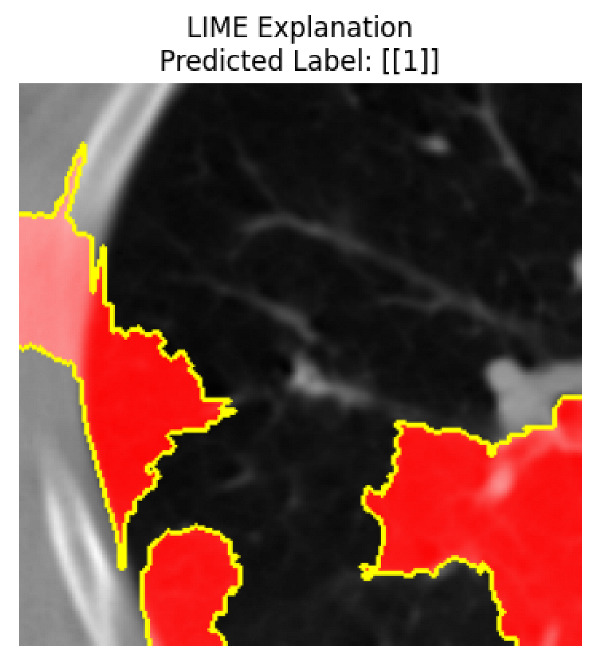
	Grad-CAM	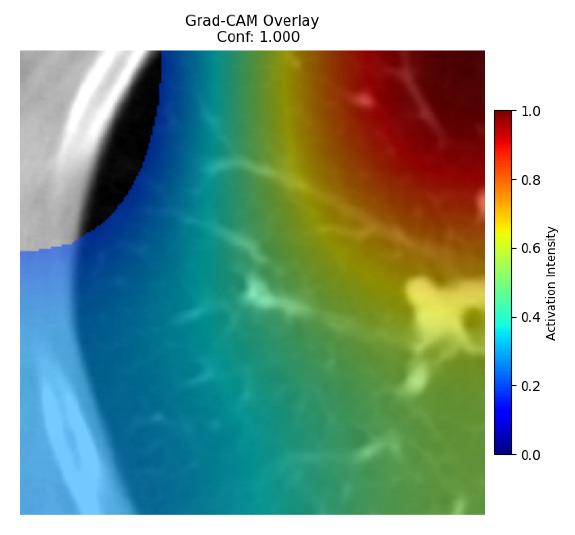	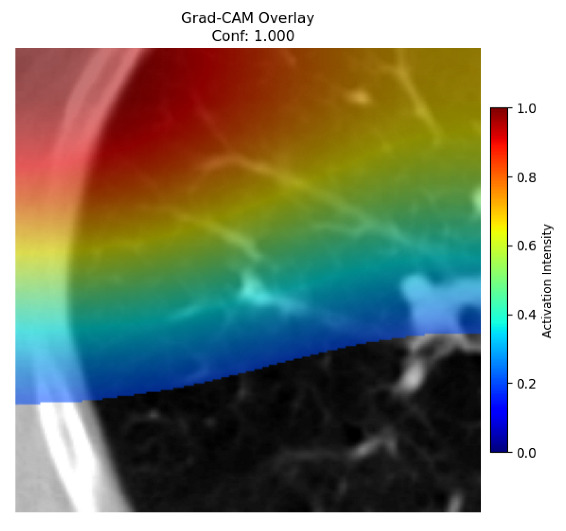	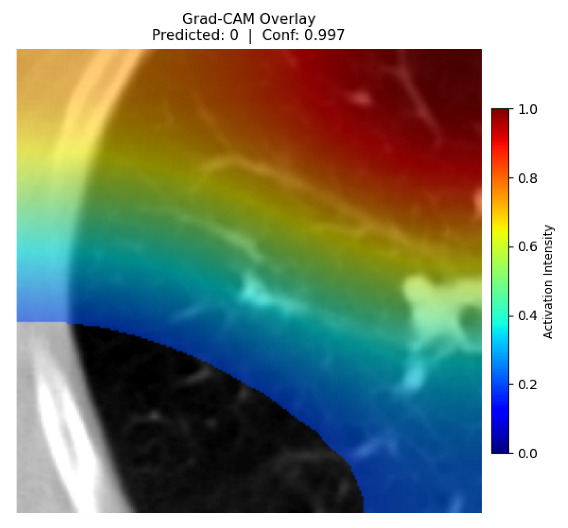	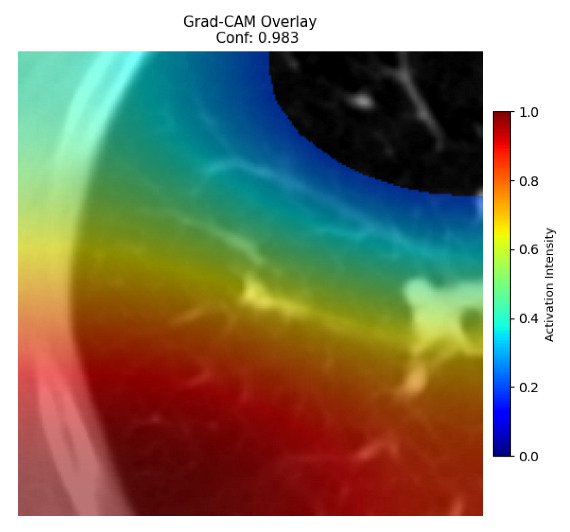

## Data Availability

The experiments and findings of this study are supported by data from publicly available datasets found in the following repositories: 1. LIDC-IDRI dataset at LIDC-IDRI (https://www.cancerimagingarchive.net/collection/lidc-idri/, accessed on 10 May 2026). 2. CT Scan Images for Lung Cancer at: CT Scan Images (https://www.kaggle.com/datasets/dishantrathi20/ct-scan-images-for-lung-cancer, accessed on 10 May 2026). 3. Lung Cancer Dataset at: lung-cancer-dataset (https://www.kaggle.com/datasets/jayaprakashpondy/lung-cancer-dataset, accessed on 10 May 2026). 4. Chest CT-Scan images Dataset at: chest-ctscan-images (https://www.kaggle.com/datasets/mohamedhanyyy/chest-ctscan-images, accessed on 10 May 2026). 5. The IQ-OTH/NCCD Dataset at: the-iqothnccd-lung-cancer-dataset (https://www.kaggle.com/hamdallak/the-iqothnccd-lung-cancer-dataset/metadata, accessed on 10 May 2026). 6. DLCTLUNGDetectNet-Lung Tumor Dataset at: dlctlungdetectnet-lung-tumor-dataset (https://www.kaggle.com/datasets/harshaldharpure/dlctlungdetectnet-lung-tumor-dataset, accessed on 10 May 2026). **Datasets Used in the Experiments:** The datasets were prepared and made publicly available at the following link: Six CT Dataset for Lung Cancer (https://www.kaggle.com/datasets/amirabouamrane/six-ct-dataset-for-lung-cancer, accessed on 10 May 2026).
